# Evaluation of a Patient With Non-Myoinvasive Uterine Serous Carcinoma Confined to a Polyp and Positive Peritoneal Washings With Somatic ARHGAP35 and KRAS Mutations

**DOI:** 10.7759/cureus.26663

**Published:** 2022-07-08

**Authors:** Sierra M Silverwood, Amir Lagstein, John I Risinger, Gregory Gressel

**Affiliations:** 1 Medical Education, Michigan State University College of Human Medicine, Grand Rapids, USA; 2 Pathology, Spectrum Health Medical Group, Grand Rapids, USA; 3 Obstetrics, Gynecology and Reproductive Biology, Spectrum Health Medical Group, Grand Rapids, USA

**Keywords:** gynaecologic oncology, histopathology examination, somatic mutations, endometrial carcinoma, peritoneal cytology

## Abstract

Currently, the application of peritoneal washings as a diagnostic tool for endometrial cancer staging is not well defined. The case described aims to highlight the current ambiguity surrounding the use of peritoneal washings in clinical practice.

A 69-year-old G3P3003 presented to her gynecologist with complaints of new-onset heavy vaginal bleeding. The patient sought an endometrial biopsy, which suggested serous endometrial intraepithelial carcinoma (EIC) focally suspicious for invasive carcinoma, with the involvement of polyps. Based on these results, a robotic-assisted total laparoscopic hysterectomy, bilateral salpingo-oophorectomy, bilateral sentinel lymph node dissection, and omentectomy were performed. Results from her final pathology exhibited a stage IA uterine serous carcinoma (USC) involving a polyp (4.2 cm in greatest dimension) with no myometrial or lymphovascular invasion, but washings were positive for adenocarcinoma. Based on her family history of malignancy, the patient underwent germline panel testing. The patient’s somatic tumor testing demonstrated proficient DNA mismatch repair status, microsatellite stability, low tumor mutational burden (4 mut/Mb), low loss of heterozygosity (9%), amplification of the ERBB2 (HER2/neu) gene by both immunohistochemistry (3+, 20% positive) and fluorescence in-situ hybridization. Her tumor also had weakly positive estrogen receptor expression (1+, 10% positive); furthermore, some pathogenic variants in KRAS (c.37G>T), PIK3CA (c.263G>A), and TP53 (c.743G>A) were identified. Given the incongruent findings found with the positive peritoneal washing and negative lymph node involvement in addition to molecular testing, management for this patient was unclear.

Ultimately, this case highlights a number of advances within the field of gynecological oncology but also emphasizes the persistent ambiguity and incongruency in the management of patients with early-stage high-risk histologies. Moving forward it will become increasingly important to be able to develop a more standardized process to assess how these diagnostic tools should inform prognosis and treatment plans.

## Introduction

Endometrial cancer represents the most common gynecologic cancer in the United States with around 66,570 new cases estimated to be diagnosed in 2021 [1]. Only about 10% of endometrial cancers are uterine serous carcinomas (USC), but they make up around 40% of deaths [2]. USC is high-grade by definition and more aggressive than most endometrioid endometrial adenocarcinomas [2]. USC has a propensity to invade local lymphatics and other intra-peritoneal structures, sometimes even in the absence of identifiable myometrial invasion, which is why many women are diagnosed with advanced-stage disease and have relatively poorer prognoses compared to women with endometrioid adenocarcinoma [3].

USC, like all endometrial cancers, is staged using the FIGO (Fédération Internationale de Gynécologie et d’Obstétrique) staging system which was revised in 2009. Prior to this time, peritoneal washings were considered an important component of endometrial cancer staging, in order to rule out the presence of malignant cells in the peritoneal cavity. Numerous studies questioned the significance of peritoneal cytology and upon investigation found little to no evidence that it was an independent prognostic factor [4-6]. These findings led to the 2009 revision of the FIGO staging system, removing peritoneal washings from the staging guidelines [7]. Despite this revision, providers have continued to use peritoneal washings as part of their diagnostic evaluation, and more recent studies have found positive results correlating with poorer prognosis [8-12]. While peritoneal cytology does not currently affect staging, FIGO, the American Joint Committee on Cancer, and the National Comprehensive Cancer Network continue to recommend obtaining washings because positive cytology may add to the effect of other poor prognostic factors and impact treatment decision-making [13].

We also have a new molecular understanding of USC based in part on work published by The Cancer Genome Atlas Network (TCGA) [14]. Where previously endometrial cancer was classified into two subgroups defined by clinical and pathologic features, comprehensive profiling by TCGA classified four separate endometrial cancer genomic signatures that could augment or replace this dualistic model and individualize treatment decision-making [15,16]. In the TCGA study, serous tumors were characterized by frequent mutations in TP53, extensive copy number alterations, and few methylation changes [14]. KRAS mutations were not common in this group (only 1%) of all USC cases whereas they were much more common in endometrioid cancer.

The following report demonstrates an interesting case of a woman with USC who had positive peritoneal washings despite non-invasive disease and was found on comprehensive tumor profiling to have a number of somatic tumor mutations which may have contributed to the aggressiveness of her disease.

## Case presentation

A 69-year-old G3P3003 presented to her gynecologist with complaints of new-onset heavy vaginal bleeding. She denied abdominal pain, dysuria, urinary frequency or urgency, weight loss, fevers, or recent injury or trauma. This was her first episode of bleeding since she became menopausal in her mid-50s. Her past medical history was significant for three spontaneous vaginal deliveries. She also had HER2/neu positive ductal carcinoma for which she had a lumpectomy and completed 10 years of anastrozole suppression and was in disease remission. Notably, the patient had a strong family history of cancer: the patient’s mother had breast and colon cancer, maternal grandmother had both breast and stomach cancer, maternal grandfather had pancreatic cancer, and a cousin had prostate cancer. Other than obesity (with a BMI of 36), she reported no other relevant medical history.

Her gynecologist performed an endometrial biopsy demonstrating at least serous endometrial intraepithelial carcinoma (EIC) focally suspicious for invasive carcinoma, with involvement of polyps. She had a normal CA125 (17.2 U/mL) and a CT scan of her chest abdomen and pelvis which showed a low-attenuating lesion in the central portion of the uterus but no lymphadenopathy or other evidence of metastatic disease. Based on these results, she was referred to a gynecologic oncologist who performed a robotic-assisted total laparoscopic hysterectomy, bilateral salpingo-oophorectomy, bilateral sentinel lymph node dissection, and omentectomy.

**Figure 1:**
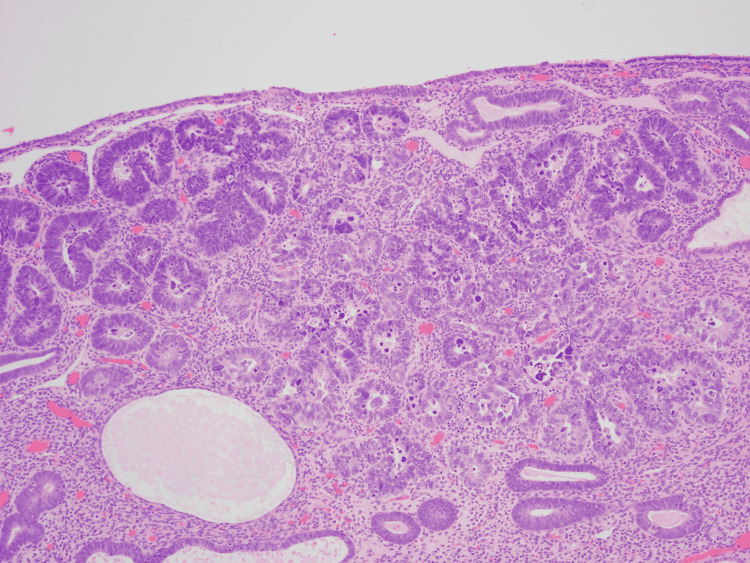
Hematoxylin and eosin stain demonstrating endometrial intraepithelial carcinoma involving a polyp. Original magnification x 100.

**Figure 2:**
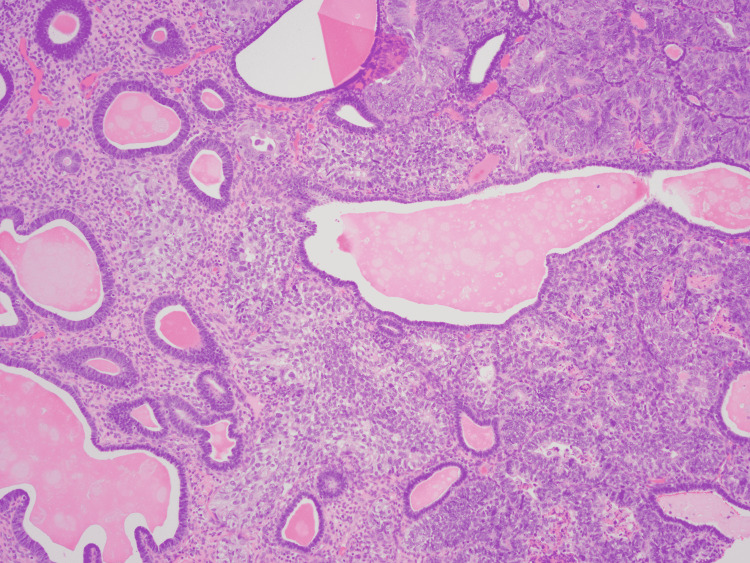
Hematoxylin and eosin stain demonstrating microscopic stromal invasion of the polyp by uterine serous carcinoma. Original magnification x 100.

**Figure 3:**
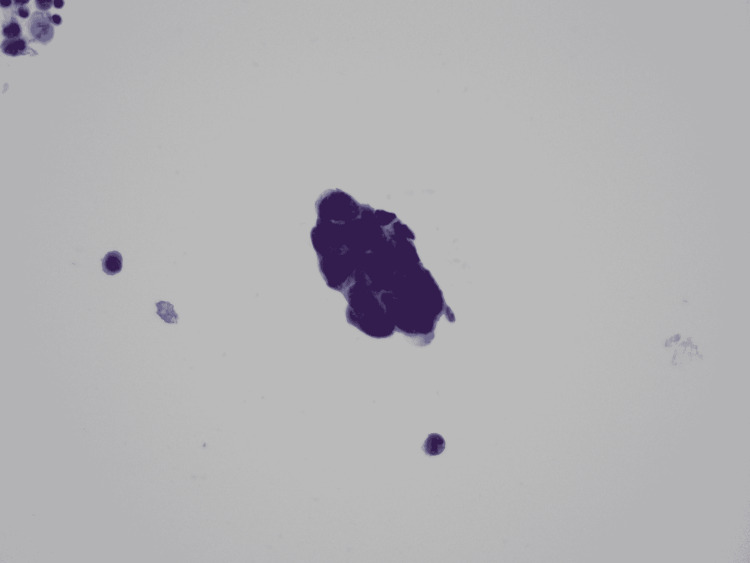
Cytology from pelvic washings demonstrating positivity for adenocarcinoma. Original magnification x 400.

Her final pathology results showed a stage IA USC involving a polyp (4.2 cm in greatest dimension) with no myometrial invasion, no lymphovascular invasion, and three negative sentinel lymph nodes but washings positive for adenocarcinoma (Figures 1-3). Her immunohistochemical tests demonstrated intact expression of DNA mismatch repair proteins (MLH1, MSH2, MSH6, PMS2).

Based on her family history of malignancy, the patient underwent germline panel testing of 47 genes frequently associated with hereditary cancers via the Invitae Common Hereditary Cancer Panel (https://www.invitae.com/). Other than a variant of uncertain significance in BARD1 (c.1409A>G; p.ASN470Ser), she had no deleterious germline mutations identified. Additionally, an archival tumor block was sent for Caris Molecular Intelligence testing. This tumor profiling strategy combines whole-exome sequencing, whole transcriptomic sequencing, and immunohistochemistry to examine cancer-type relevant biomarkers. Details regarding this assay can be found at Caris Life Sciences: Comprehensive Molecular Profiling (https://www.carismolecularintelligence.com/comprehensivetumorprofiling.)

The patient’s somatic tumor testing was notable for proficient DNA mismatch repair status, microsatellite stability, low tumor mutational burden (4 mut/Mb), low loss of heterozygosity (9%), amplification of the ERBB2 (Her2/Neu) gene by both immunohistochemistry (3+, 20% positive) and fluorescence in-situ hybridization. Her tumor also had weakly positive estrogen receptor expression (1+, 10% positive) but negative progesterone receptor staining (0%). There were notable somatic pathogenic variants in KRAS (c.37G>T), PIK3CA (c.263G>A), and TP53 (c.743G>A).

Given the results from the molecular profiling along with the incongruent findings found with the positive peritoneal washing and negative lymph node involvement, the steps to manage this patient were not well defined. Ultimately, the patient was presented at the tumor board where it was decided that she would proceed with six cycles of carboplatin and paclitaxel followed by cuff brachytherapy. This case, however, created an opportunity to discuss the current space for peritoneal washings in informing treatment regimens, and the way molecular profiling can help direct the management of patients that present with early-stage USC.

## Discussion

Our understanding of the prognostic value of peritoneal cytology has been evolving since the 2009 FIGO staging modifications [11]. An estimated 11% of women who undergo staging surgery for endometrial cancer will have positive peritoneal cytology, most commonly in the presence of extra-uterine disease. The prognostic significance of isolated peritoneal cytology in the absence of other risk factors is controversial and therefore it is not often used as a high-risk criterion in the formulation of adjuvant treatment planning.

Several recent studies have found peritoneal cytology to be an important prognostic for survival in women with early-stage endometrial cancer [9,10]. One study conducted by Matsuo et al. found that in a population of 1668 women with early-stage endometrioid cancer, the presence of abnormal peritoneal cytology (11% of their cohort) was independently associated with decreased disease-free survival (HR 3.07, P<0.001) and cause-specific survival (HR 3.42, P = 0.008) [9]. Another recent study out of Michigan examined a cohort of 148 women with USC and found that 22% of the cohort had positive washings. The presence of malignant cytology in this study was significantly associated with recurrence and overall survival (HR 2.09, 95% CI [1.19-3.68]) [17]. A large Surveillance, Epidemiology, and End Results database study of over 14,000 patients with uterine cancer identified that five-year disease-specific survival for women with negative cytology was significantly better than those with positive cytology (95.1% vs 80.8% in endometrioid adenocarcinoma and 78.0% vs 50.4% in clear cell or serous carcinoma) [18]. Because of this, some experts have proposed a new staging schema with stage-specific incorporation of malignant peritoneal cytology [19].

These findings become especially relevant when evaluating a patient with peritoneal washing results that do not align with the degree of myometrial invasion, presence of lymphovascular invasion, or of nodal involvement. In situations like this case, if abnormal peritoneal cytology is a prognostic factor for decreased survival, as has been suggested in these studies, it may be necessary to approach treatment more aggressively [8].

We know that women with the completely staged disease and no residual tumor in their hysterectomy specimen after dilation and curettage or hysteroscopic resection do not often experience recurrence regardless of adjuvant therapy. However, multiple studies have demonstrated that women with early-stage USC experience improved disease-free survival and overall survival with platinum-based chemotherapy [2,20]. It remains unclear if women with early-stage high-risk histologies with positive pelvic washings would benefit from aggressive adjuvant therapy in the absence of other risk factors for recurrence. Mysona et al. published a case series of 1751 patients with stage IA USC in which 7% had malignant cytology [21]. The presence of positive peritoneal cytology was associated in multivariate analysis with an increased hazard of death (HR 2.62, 95% CI [1.79-3.82]). This group also produced a nomogram based on Cox regression analysis to help predict which women with stage IA USC would benefit from adjuvant chemotherapy. However, in their backward recursive elimination, peritoneal cytology was left out of their nomogram. Based on her age and tumor size, this Cox-based nomogram would have classified her case as “moderate risk” with a slightly greater than 70% probability of surviving at five years. In their group of moderate-risk women, chemotherapy improved five-year survival by 9%. They also published a clinical calculator based on a random survival forest approach which would have given our patient a score of 8.45, which suggests she would not have benefited from chemotherapy. These examples highlight the difficulty of risk-stratification of women with early-stage high-risk histologies and determining for whom adjuvant treatment is most appropriate.

In addition to reassessing the role of peritoneal washings in informing this patient’s treatment, it is important to take into consideration the results of her molecular testing. Recent advancements in molecular testing in this patient population have created opportunities for novel treatment modalities [22]. One area of current exploration is the role of adding trastuzumab to adjuvant chemotherapy in HER2+ serous cancers. Recent studies have revealed HER2/neu amplification to be present in 18-42% of USC patients [23]. These results suggest that HER2 therapies based on monoclonal antibodies or tyrosine kinase inhibitors could be an effective new approach to treating patients with USC [24]. Trastuzumab is a monoclonal antibody that specifically targets HER2/neu [25]. Early studies investigating trastuzumab combined with carboplatin and paclitaxel chemotherapy in advanced and recurrent USC patients overexpressing HER2/neu have shown promising results. In a landmark phase II trial of women with stage III-IV or recurrent HER2 over-expressing uterine serous carcinomas, the addition of trastuzumab to carboplatin and paclitaxel as adjuvant treatment improved overall survival by over five months (HR 0.58; 95% [CI 0.34-0.99]) [26]. More recent studies looking specifically at the benefit of this approach in early-stage HER2/neu positive patients found patients who received trastuzumab also showed improved progression-free survival. The greatest benefit, however, was still identified in patients with stage III-IV disease [23]. While only a handful of studies have been conducted in early-stage patients, they are starting to show promising results [23,27]. It remains unclear if the cost of this medication would be justifiable (or even beneficial) in women with early-stage, HER2 overexpressing disease.

This patient also had TP53 and PIK3CA mutations with a low mutation frequency and microsatellite stable. These results are not unusual for this patient population but do have implications on prognosis and treatment [28]. In one study it was found that individuals with both TP53 and PIK3CA mutations had worse survival prognoses than those with only TP53 mutation [29]. PIK3CA mutations, specifically, have also been implicated in the efficacy of HER2-targeted therapy. Despite the promise of HER2 targeted therapy, PIK3CA mutations may serve as a mechanism by which resistance to trastuzumab is acquired. Black et al found a statistically significant increase in the prevalence of acquired trastuzumab resistance in the presence of PIK3CA mutations using mouse models [30]. Nevertheless, both these mutations, TP53 and PIK3CA, offer promising alternative therapeutic targets for USC. Currently, clinical trials are being conducted to assess the efficacy of both WEE1, a key cell cycle regular, and PI3K/AKT/mTOR inhibitors. Both studies have already begun to report promising results when using these targeted therapies [25,31]. Overall, these studies highlight the significance of these molecular markers as both prognostic factors and therapeutic targets, especially in early-stage HER2+ USC patients.

This patient’s tumor also had several other mutations worth noting including a truncation in ARHGAP35 and a very rarely occurring KRAS variant. Mutations in ARHGAP35 were found in 18% of the uterine serous cancers in TCGA making it one of the most frequently mutated genes in this cancer type [32,33]. Whereas ARHGAP35 mutations often occur simultaneously with TP53 mutations in USC, molecular data from carcinosarcomas demonstrate these mutations occur more commonly in tumors without TP53 mutations [34]. The role of ARHGAP35 mutations in carcinogenesis is still not completely understood [35]. This gene is associated with the regulation of RhoA, a transforming protein associated with the regulation of the cytoskeleton [36]. Despite the emergence of ARHGAP35 mutations as a new major cancer gene, few studies have investigated its implication for the prognosis and treatment of USC. The ARHGAP35 mutation in this study was a truncation (p.R617) suggesting loss of ARHGAP35 normal functions.

A KRAS G13C mutation was also identified in this patient’s tumor molecular data. While KRAS mutation is common in endometrioid endometrial cancers it is rare in uterine serous tumors occurring in less than 3% of cases. This particular mutation was not seen in any other serous case. Despite the rarity of this mutation, it could, however, make this patient eligible for pan-RAS inhibitors which target KRAS mutations. No studies, however, have examined this specific mutation in this population; therefore, more investigation is required in order to understand how these results should inform prognosis and treatment. In addition to ARHGAP35 and KRAS genes, several other mutations were noted including those in BARD1 (also noted in the hereditary cancer germline analysis described above), YYAP1 (YAP), POLH, and MLH3 among others. The complete list of detected variants, amplifications, and fusion events is described in the supplemental table provided in the appendix.

## Conclusions

Ultimately, this case highlights some exciting changes that have occurred in our field over the past 10 years, but also hints at persistent ambiguity and confusion in managing patients with early-stage high-risk histologies. Moving forward it will become increasingly important to be able to, firstly, integrate the results of a patient’s histopathology with their molecular testing, secondly, reconcile how these findings impact prognosis, and thirdly, develop treatment plans based on those results.

## Appendices

**Table 1:** Complete list of detected variants, amplifications, and fusion events identified in the patient’s tumor

Biomarker	Method	Analyte Descrip	Result	Variant Detail	Variant Frequency(%)
ABCB11	Seq	DNA-Tumor	Mutation Not Detected		
ABL1	Seq	DNA-Tumor	Mutation Not Detected		
ABL1	Seq	RNA-Tumor	Fusion Not Detected		
ABL2	CNA-Seq	DNA-Tumor	Amplification Not Detected		
ABL2	Seq	DNA-Tumor	Mutation Not Detected		
ABRAXAS1	Seq	DNA-Tumor	Mutation Not Detected		
ACD	Seq	DNA-Tumor	Mutation Not Detected		
ACKR3	Seq	DNA-Tumor	Mutation Not Detected		
ACSL3	CNA-Seq	DNA-Tumor	Amplification Not Detected		
ACSL3	Seq	DNA-Tumor	Mutation Not Detected		
ACSL6	CNA-Seq	DNA-Tumor	Amplification Not Detected		
ACSL6	Seq	DNA-Tumor	Mutation Not Detected		
ACVR1	Seq	DNA-Tumor	Mutation Not Detected		
ACVR1B	Seq	DNA-Tumor	Indeterminate		
ADGRA2	Seq	DNA-Tumor	Mutation Not Detected		
AFDN	CNA-Seq	DNA-Tumor	Amplification Not Detected		
AFDN	Seq	DNA-Tumor	Mutation Not Detected		
AFF1	CNA-Seq	DNA-Tumor	Amplification Not Detected		
AFF1	Seq	DNA-Tumor	Mutation Not Detected		
AFF3	CNA-Seq	DNA-Tumor	Amplification Not Detected		
AFF3	Seq	DNA-Tumor	Mutation Not Detected		
AIP	Seq	DNA-Tumor	Mutation Not Detected		
AJUBA	Seq	DNA-Tumor	Mutation Not Detected		
AKT1	Seq	DNA-Tumor	Mutation Not Detected		
AKT2	CNA-Seq	DNA-Tumor	Amplification Not Detected		
AKT2	Seq	DNA-Tumor	Mutation Not Detected		
AKT3	CNA-Seq	DNA-Tumor	Amplification Not Detected		
AKT3	Seq	DNA-Tumor	Mutation Not Detected		
AKT3	Seq	RNA-Tumor	Fusion Not Detected		
ALK	CNA-Seq	DNA-Tumor	Amplification Not Detected		
ALK	Seq	DNA-Tumor	Mutation Not Detected		
ALK	Seq	RNA-Tumor	Fusion Not Detected		
ALOX12B	Seq	DNA-Tumor	Mutation Not Detected		
AMER1	Seq	DNA-Tumor	Mutation Not Detected		
ANKRD26	Seq	DNA-Tumor	Indeterminate		
APC	CNA-Seq	DNA-Tumor	Amplification Not Detected		
APC	Seq	DNA-Tumor	Mutation Not Detected		
APLNR	Seq	DNA-Tumor	Mutation Not Detected		
AR	Seq	DNA-Tumor	Mutation Not Detected		
AR	Seq	RNA-Tumor	Variant Transcript Not Detected		
ARAF	Seq	DNA-Tumor	Mutation Not Detected		
ARFRP1	Seq	DNA-Tumor	Mutation Not Detected		
ARHGAP26	Seq	RNA-Tumor	Fusion Not Detected		
ARHGAP35	Seq	DNA-Tumor	Unclassified Variant	Exon 1 | p.R617	41
ARHGEF12	Seq	DNA-Tumor	Indeterminate		
ARID1A	CNA-Seq	DNA-Tumor	Amplification Not Detected		
ARID1A	Seq	DNA-Tumor	Mutation Not Detected		
ARID1B	Seq	DNA-Tumor	Mutation Not Detected		
ARID2	CNA-Seq	DNA-Tumor	Amplification Not Detected		
ARID2	Seq	DNA-Tumor	Mutation Not Detected		
ARID5B	Seq	DNA-Tumor	Mutation Not Detected		
ARNT	CNA-Seq	DNA-Tumor	Amplification Not Detected		
ARNT	Seq	DNA-Tumor	Mutation Not Detected		
ASPSCR1	Seq	DNA-Tumor	Unclassified Variant	Exon 13 | p.A458T	56
ASXL1	CNA-Seq	DNA-Tumor	Amplification Not Detected		
ASXL1	Seq	DNA-Tumor	Mutation Not Detected		
ASXL2	Seq	DNA-Tumor	Indeterminate		
ATF1	CNA-Seq	DNA-Tumor	Amplification Not Detected		
ATF1	Seq	DNA-Tumor	Mutation Not Detected		
ATIC	CNA-Seq	DNA-Tumor	Amplification Not Detected		
ATIC	Seq	DNA-Tumor	Mutation Not Detected		
ATM	CNA-Seq	DNA-Tumor	Amplification Not Detected		
ATM	Seq	DNA-Tumor	Mutation Not Detected		
ATP1A1	CNA-Seq	DNA-Tumor	Amplification Not Detected		
ATP1A1	Seq	DNA-Tumor	Mutation Not Detected		
ATR	CNA-Seq	DNA-Tumor	Amplification Not Detected		
ATR	Seq	DNA-Tumor	Mutation Not Detected		
ATRX	Seq	DNA-Tumor	Mutation Not Detected		
AURKA	Seq	DNA-Tumor	Indeterminate		
AURKB	CNA-Seq	DNA-Tumor	Amplification Not Detected		
AURKB	Seq	DNA-Tumor	Mutation Not Detected		
AXIN1	Seq	DNA-Tumor	Mutation Not Detected		
AXIN2	Seq	DNA-Tumor	Mutation Not Detected		
AXL	Seq	DNA-Tumor	Mutation Not Detected		
AXL	Seq	RNA-Tumor	Fusion Not Detected		
B2M	Seq	DNA-Tumor	Mutation Not Detected		
BAP1	CNA-Seq	DNA-Tumor	Amplification Not Detected		
BAP1	Seq	DNA-Tumor	Mutation Not Detected		
BARD1	CNA-Seq	DNA-Tumor	Amplification Not Detected		
BARD1	Seq	DNA-Tumor	Unclassified Variant	Exon 6 | p.N470S	48
BCL10	Seq	DNA-Tumor	Mutation Not Detected		
BCL11A	CNA-Seq	DNA-Tumor	Amplification Not Detected		
BCL11A	Seq	DNA-Tumor	Mutation Not Detected		
BCL11B	Seq	DNA-Tumor	Mutation Not Detected		
BCL2	Seq	DNA-Tumor	Mutation Not Detected		
BCL2L1	Seq	DNA-Tumor	Mutation Not Detected		
BCL2L11	CNA-Seq	DNA-Tumor	Amplification Not Detected		
BCL2L11	Seq	DNA-Tumor	Mutation Not Detected		
BCL2L12	Seq	DNA-Tumor	Indeterminate		
BCL2L2	Seq	DNA-Tumor	Mutation Not Detected		
BCL3	CNA-Seq	DNA-Tumor	Amplification Not Detected		
BCL3	Seq	DNA-Tumor	Indeterminate		
BCL6	CNA-Seq	DNA-Tumor	Amplification Not Detected		
BCL6	Seq	DNA-Tumor	Mutation Not Detected		
BCL9	CNA-Seq	DNA-Tumor	Amplification Not Detected		
BCL9	Seq	DNA-Tumor	Mutation Not Detected		
BCOR	Seq	DNA-Tumor	Mutation Not Detected		
BCORL1	Seq	DNA-Tumor	Mutation Not Detected		
BCR	Seq	RNA-Tumor	Fusion Not Detected		
BIRC3	Seq	DNA-Tumor	Indeterminate		
BLM	CNA-Seq	DNA-Tumor	Amplification Not Detected		
BLM	Seq	DNA-Tumor	Mutation Not Detected		
BMPR1A	CNA-Seq	DNA-Tumor	Amplification Not Detected		
BMPR1A	Seq	DNA-Tumor	Mutation Not Detected		
BRAF	CNA-Seq	DNA-Tumor	Amplification Not Detected		
BRAF	Seq	DNA-Tumor	Mutation Not Detected		
BRAF	Seq	RNA-Tumor	Fusion Not Detected		
BRCA1	CNA-Seq	DNA-Tumor	Amplification Not Detected		
BRCA1	Seq	DNA-Tumor	Mutation Not Detected		
BRCA2	CNA-Seq	DNA-Tumor	Amplification Not Detected		
BRCA2	Seq	DNA-Tumor	Mutation Not Detected		
BRD3	Seq	DNA-Tumor	Mutation Not Detected		
BRD3	Seq	RNA-Tumor	Fusion Not Detected		
BRD4	Seq	DNA-Tumor	Mutation Not Detected		
BRD4	Seq	RNA-Tumor	Fusion Not Detected		
BRIP1	CNA-Seq	DNA-Tumor	Amplification Not Detected		
BRIP1	Seq	DNA-Tumor	Mutation Not Detected		
BTG1	Seq	DNA-Tumor	Mutation Not Detected		
BTG2	Seq	DNA-Tumor	Mutation Not Detected		
BTK	Seq	DNA-Tumor	Mutation Not Detected		
BUB1B	Seq	DNA-Tumor	Mutation Not Detected		
C15orf65	Seq	DNA-Tumor	Mutation Not Detected		
CACNA1D	CNA-Seq	DNA-Tumor	Amplification Not Detected		
CACNA1D	Seq	DNA-Tumor	Mutation Not Detected		
CALR	CNA-Seq	DNA-Tumor	Amplification Not Detected		
CALR	Seq	DNA-Tumor	Mutation Not Detected		
CAMTA1	CNA-Seq	DNA-Tumor	Amplification Not Detected		
CAMTA1	Seq	DNA-Tumor	Indeterminate		
CARD11	CNA-Seq	DNA-Tumor	Amplification Not Detected		
CARD11	Seq	DNA-Tumor	Mutation Not Detected		
CARS	CNA-Seq	DNA-Tumor	Amplification Not Detected		
CARS	Seq	DNA-Tumor	Mutation Not Detected		
CASP8	CNA-Seq	DNA-Tumor	Amplification Not Detected		
CASP8	Seq	DNA-Tumor	Mutation Not Detected		
CBFA2T3	Seq	DNA-Tumor	Mutation Not Detected		
CBFB	CNA-Seq	DNA-Tumor	Amplification Not Detected		
CBFB	Seq	DNA-Tumor	Mutation Not Detected		
CBL	CNA-Seq	DNA-Tumor	Amplification Not Detected		
CBL	Seq	DNA-Tumor	Mutation Not Detected		
CBLB	Seq	DNA-Tumor	Mutation Not Detected		
CCDC6	CNA-Seq	DNA-Tumor	Amplification Not Detected		
CCDC6	Seq	DNA-Tumor	Mutation Not Detected		
CCND1	CNA-Seq	DNA-Tumor	Amplification Not Detected		
CCND1	Seq	DNA-Tumor	Mutation Not Detected		
CCND2	CNA-Seq	DNA-Tumor	Amplification Not Detected		
CCND2	Seq	DNA-Tumor	Mutation Not Detected		
CCND3	CNA-Seq	DNA-Tumor	Amplification Not Detected		
CCND3	Seq	DNA-Tumor	Mutation Not Detected		
CCNE1	CNA-Seq	DNA-Tumor	Intermediate		
CCNE1	Seq	DNA-Tumor	Mutation Not Detected		
CD22	Seq	DNA-Tumor	Mutation Not Detected		
CD274 (PD-L1)	CNA-Seq	DNA-Tumor	Amplification Not Detected		
CD274 (PD-L1)	Seq	DNA-Tumor	Mutation Not Detected		
CD70	Seq	DNA-Tumor	Mutation Not Detected		
CD74	CNA-Seq	DNA-Tumor	Amplification Not Detected		
CD74	Seq	DNA-Tumor	Mutation Not Detected		
CD79A	CNA-Seq	DNA-Tumor	Amplification Not Detected		
CD79A	Seq	DNA-Tumor	Mutation Not Detected		
CD79B	Seq	DNA-Tumor	Mutation Not Detected		
CDC73	CNA-Seq	DNA-Tumor	Amplification Not Detected		
CDC73	Seq	DNA-Tumor	Mutation Not Detected		
CDH1	Seq	DNA-Tumor	Mutation Not Detected		
CDH11	CNA-Seq	DNA-Tumor	Amplification Not Detected		
CDH11	Seq	DNA-Tumor	Mutation Not Detected		
CDH23	Seq	DNA-Tumor	Mutation Not Detected		
CDK12	Seq	DNA-Tumor	Mutation Not Detected		
CDK4	CNA-Seq	DNA-Tumor	Amplification Not Detected		
CDK4	Seq	DNA-Tumor	Mutation Not Detected		
CDK6	CNA-Seq	DNA-Tumor	Amplification Not Detected		
CDK6	Seq	DNA-Tumor	Mutation Not Detected		
CDK8	CNA-Seq	DNA-Tumor	Amplification Not Detected		
CDK8	Seq	DNA-Tumor	Mutation Not Detected		
CDKN1A	Seq	DNA-Tumor	Mutation Not Detected		
CDKN1B	CNA-Seq	DNA-Tumor	Amplification Not Detected		
CDKN1B	Seq	DNA-Tumor	Mutation Not Detected		
CDKN1C	Seq	DNA-Tumor	Mutation Not Detected		
CDKN2A	CNA-Seq	DNA-Tumor	Amplification Not Detected		
CDKN2A	Seq	DNA-Tumor	Mutation Not Detected		
CDKN2B	Seq	DNA-Tumor	Mutation Not Detected		
CDKN2C	Seq	DNA-Tumor	Mutation Not Detected		
CDX2	CNA-Seq	DNA-Tumor	Amplification Not Detected		
CDX2	Seq	DNA-Tumor	Mutation Not Detected		
CEBPA	Seq	DNA-Tumor	Mutation Not Detected		
CHD2	Seq	DNA-Tumor	Indeterminate		
CHD4	Seq	DNA-Tumor	Mutation Not Detected		
CHEK1	CNA-Seq	DNA-Tumor	Amplification Not Detected		
CHEK1	Seq	DNA-Tumor	Mutation Not Detected		
CHEK2	CNA-Seq	DNA-Tumor	Amplification Not Detected		
CHEK2	Seq	DNA-Tumor	Mutation Not Detected		
CHIC2	CNA-Seq	DNA-Tumor	Amplification Not Detected		
CHIC2	Seq	DNA-Tumor	Mutation Not Detected		
CHN1	Seq	DNA-Tumor	Indeterminate		
CIC	CNA-Seq	DNA-Tumor	Amplification Not Detected		
CIC	Seq	DNA-Tumor	Unclassified Variant	Exon 11 | p.R934L	55
CIITA	Seq	DNA-Tumor	Indeterminate		
CLTCL1	CNA-Seq	DNA-Tumor	Amplification Not Detected		
CLTCL1	Seq	DNA-Tumor	Mutation Not Detected		
CLYBL	Seq	DNA-Tumor	Mutation Not Detected		
CNBP	CNA-Seq	DNA-Tumor	Amplification Not Detected		
CNBP	Seq	DNA-Tumor	Mutation Not Detected		
CNOT3	Seq	DNA-Tumor	Mutation Not Detected		
COX6C	Seq	DNA-Tumor	Mutation Not Detected		
CREB1	Seq	DNA-Tumor	Mutation Not Detected		
CREB3L1	Seq	DNA-Tumor	Mutation Not Detected		
CREB3L2	CNA-Seq	DNA-Tumor	Amplification Not Detected		
CREB3L2	Seq	DNA-Tumor	Unclassified Variant	Exon 11 | p.V488M	5
CREBBP	CNA-Seq	DNA-Tumor	Amplification Not Detected		
CREBBP	Seq	DNA-Tumor	Mutation Not Detected		
CRKL	CNA-Seq	DNA-Tumor	Amplification Not Detected		
CRKL	Seq	DNA-Tumor	Mutation Not Detected		
CRLF2	Seq	DNA-Tumor	Indeterminate		
CRTC1	Seq	DNA-Tumor	Mutation Not Detected		
CRTC3	CNA-Seq	DNA-Tumor	Amplification Not Detected		
CRTC3	Seq	DNA-Tumor	Mutation Not Detected		
CSF1R	CNA-Seq	DNA-Tumor	Amplification Not Detected		
CSF1R	Seq	DNA-Tumor	Mutation Not Detected		
CSF3R	Seq	DNA-Tumor	Mutation Not Detected		
CTCF	CNA-Seq	DNA-Tumor	Amplification Not Detected		
CTCF	Seq	DNA-Tumor	Mutation Not Detected		
CTLA4	Seq	DNA-Tumor	Mutation Not Detected		
CTNNA1	CNA-Seq	DNA-Tumor	Amplification Not Detected		
CTNNA1	Seq	DNA-Tumor	Mutation Not Detected		
CTNNB1	CNA-Seq	DNA-Tumor	Amplification Not Detected		
CTNNB1	Seq	DNA-Tumor	Mutation Not Detected		
CUL3	Seq	DNA-Tumor	Mutation Not Detected		
CUL4A	Seq	DNA-Tumor	Mutation Not Detected		
CUX1	Seq	DNA-Tumor	Mutation Not Detected		
CXCR4	Seq	DNA-Tumor	Mutation Not Detected		
CYLD	CNA-Seq	DNA-Tumor	Amplification Not Detected		
CYLD	Seq	DNA-Tumor	Mutation Not Detected		
CYP17A1	Seq	DNA-Tumor	Mutation Not Detected		
CYP2D6	Seq	DNA-Tumor	Mutation Not Detected		
DAXX	CNA-Seq	DNA-Tumor	Amplification Not Detected		
DAXX	Seq	DNA-Tumor	Mutation Not Detected		
DDB2	Seq	DNA-Tumor	Mutation Not Detected		
DDIT3	Seq	DNA-Tumor	Mutation Not Detected		
DDR1	Seq	DNA-Tumor	Indeterminate		
DDR2	CNA-Seq	DNA-Tumor	Amplification Not Detected		
DDR2	Seq	DNA-Tumor	Mutation Not Detected		
DDX3X	Seq	DNA-Tumor	Mutation Not Detected		
DDX41	Seq	DNA-Tumor	Mutation Not Detected		
DDX6	CNA-Seq	DNA-Tumor	Amplification Not Detected		
DDX6	Seq	DNA-Tumor	Mutation Not Detected		
DEK	CNA-Seq	DNA-Tumor	Amplification Not Detected		
DEK	Seq	DNA-Tumor	Indeterminate		
DICER1	CNA-Seq	DNA-Tumor	Amplification Not Detected		
DICER1	Seq	DNA-Tumor	Mutation Not Detected		
DIS3	Seq	DNA-Tumor	Mutation Not Detected		
DIS3L2	Seq	DNA-Tumor	Mutation Not Detected		
DKC1	Seq	DNA-Tumor	Mutation Not Detected		
DMC1	Seq	DNA-Tumor	Mutation Not Detected		
DNA2	Seq	DNA-Tumor	Indeterminate		
DNAJB1	Seq	DNA-Tumor	Mutation Not Detected		
DNMT3A	Seq	DNA-Tumor	Mutation Not Detected		
DOT1L	Seq	DNA-Tumor	Indeterminate		
EBF1	CNA-Seq	DNA-Tumor	Amplification Not Detected		
EBF1	Seq	DNA-Tumor	Mutation Not Detected		
ECT2L	CNA-Seq	DNA-Tumor	Amplification Not Detected		
ECT2L	Seq	DNA-Tumor	Mutation Not Detected		
EED	Seq	DNA-Tumor	Mutation Not Detected		
EGFR	CNA-Seq	DNA-Tumor	Amplification Not Detected		
EGFR	Seq	DNA-Tumor	Mutation Not Detected		
EGFR	Seq	RNA-Tumor	Fusion Not Detected		
EGFRvIII	Seq	RNA-Tumor	Variant Transcript Not Detected		
EGLN1	Seq	DNA-Tumor	Mutation Not Detected		
EIF1AX	Seq	DNA-Tumor	Mutation Not Detected		
EIF4A2	Seq	DNA-Tumor	Mutation Not Detected		
ELF3	Seq	DNA-Tumor	Mutation Not Detected		
ELK4	CNA-Seq	DNA-Tumor	Amplification Not Detected		
ELK4	Seq	DNA-Tumor	Mutation Not Detected		
ELOC	Seq	DNA-Tumor	Indeterminate		
EME1	Seq	DNA-Tumor	Mutation Not Detected		
EME2	Seq	DNA-Tumor	Mutation Not Detected		
EML4	Seq	DNA-Tumor	Indeterminate		
EMSY	Seq	DNA-Tumor	Mutation Not Detected		
ENAH	Seq	RNA-Tumor	Unclassified Fusion Detected	ENAH:ACBD3 |	
EP300	CNA-Seq	DNA-Tumor	Amplification Not Detected		
EP300	Seq	DNA-Tumor	Mutation Not Detected		
EPCAM	Seq	DNA-Tumor	Mutation Not Detected		
EPHA2	Seq	DNA-Tumor	Mutation Not Detected		
EPHA3	CNA-Seq	DNA-Tumor	Amplification Not Detected		
EPHA3	Seq	DNA-Tumor	Mutation Not Detected		
EPHA5	Seq	DNA-Tumor	Mutation Not Detected		
EPHA7	Seq	DNA-Tumor	Mutation Not Detected		
EPHB1	Seq	DNA-Tumor	Mutation Not Detected		
EPHB4	Seq	DNA-Tumor	Mutation Not Detected		
ER	IHC	Protein	Positive | 1+, 10%		
ERBB2 (Her2/Neu)	CISH	DNA-Tumor	Amplified		
ERBB2 (Her2/Neu)	CNA-Seq	DNA-Tumor	Intermediate		
ERBB2 (Her2/Neu)	IHC	Protein	Positive | 3+, 20%		
ERBB2 (Her2/Neu)	Seq	DNA-Tumor	Mutation Not Detected		
ERBB3	CNA-Seq	DNA-Tumor	Amplification Not Detected		
ERBB3	Seq	DNA-Tumor	Mutation Not Detected		
ERBB4	CNA-Seq	DNA-Tumor	Amplification Not Detected		
ERBB4	Seq	DNA-Tumor	Mutation Not Detected		
ERC1	Seq	DNA-Tumor	Mutation Not Detected		
ERCC1	Seq	DNA-Tumor	Mutation Not Detected		
ERCC2	CNA-Seq	DNA-Tumor	Amplification Not Detected		
ERCC2	Seq	DNA-Tumor	Mutation Not Detected		
ERCC3	CNA-Seq	DNA-Tumor	Amplification Not Detected		
ERCC3	Seq	DNA-Tumor	Mutation Not Detected		
ERCC4	Seq	DNA-Tumor	Mutation Not Detected		
ERCC5	Seq	DNA-Tumor	Mutation Not Detected		
ERCC6	Seq	DNA-Tumor	Mutation Not Detected		
EREG	Seq	DNA-Tumor	Mutation Not Detected		
ERG	CNA-Seq	DNA-Tumor	Amplification Not Detected		
ERG	Seq	DNA-Tumor	Mutation Not Detected		
ERG	Seq	RNA-Tumor	Fusion Not Detected		
ERRFI1	Seq	DNA-Tumor	Mutation Not Detected		
ESR1	CNA-Seq	DNA-Tumor	Amplification Not Detected		
ESR1	Seq	DNA-Tumor	Mutation Not Detected		
ESR1	Seq	RNA-Tumor	Fusion Not Detected		
ETS1	Seq	DNA-Tumor	Mutation Not Detected		
ETV1	CNA-Seq	DNA-Tumor	Amplification Not Detected		
ETV1	Seq	DNA-Tumor	Mutation Not Detected		
ETV1	Seq	RNA-Tumor	Fusion Not Detected		
ETV4	Seq	DNA-Tumor	Mutation Not Detected		
ETV4	Seq	RNA-Tumor	Fusion Not Detected		
ETV5	CNA-Seq	DNA-Tumor	Amplification Not Detected		
ETV5	Seq	DNA-Tumor	Mutation Not Detected		
ETV5	Seq	RNA-Tumor	Fusion Not Detected		
ETV6	CNA-Seq	DNA-Tumor	Amplification Not Detected		
ETV6	Seq	DNA-Tumor	Mutation Not Detected		
ETV6	Seq	RNA-Tumor	Fusion Not Detected		
EWSR1	CNA-Seq	DNA-Tumor	Amplification Not Detected		
EWSR1	Seq	DNA-Tumor	Mutation Not Detected		
EWSR1	Seq	RNA-Tumor	Fusion Not Detected		
EXO1	Seq	DNA-Tumor	Mutation Not Detected		
EXT1	CNA-Seq	DNA-Tumor	Amplification Not Detected		
EXT1	Seq	DNA-Tumor	Mutation Not Detected		
EXT2	CNA-Seq	DNA-Tumor	Amplification Not Detected		
EXT2	Seq	DNA-Tumor	Mutation Not Detected		
EZH2	CNA-Seq	DNA-Tumor	Amplification Not Detected		
EZH2	Seq	DNA-Tumor	Mutation Not Detected		
EZR	CNA-Seq	DNA-Tumor	Amplification Not Detected		
EZR	Seq	DNA-Tumor	Mutation Not Detected		
FAM46C	Seq	DNA-Tumor	Mutation Not Detected		
FANCA	CNA-Seq	DNA-Tumor	Amplification Not Detected		
FANCA	Seq	DNA-Tumor	Mutation Not Detected		
FANCB	Seq	DNA-Tumor	Mutation Not Detected		
FANCC	CNA-Seq	DNA-Tumor	Amplification Not Detected		
FANCC	Seq	DNA-Tumor	Mutation Not Detected		
FANCD2	CNA-Seq	DNA-Tumor	Amplification Not Detected		
FANCD2	Seq	DNA-Tumor	Variant of Uncertain Significance	Exon 3 | p.Q26H	39
FANCE	CNA-Seq	DNA-Tumor	Amplification Not Detected		
FANCE	Seq	DNA-Tumor	Mutation Not Detected		
FANCF	Seq	DNA-Tumor	Mutation Not Detected		
FANCG	CNA-Seq	DNA-Tumor	Amplification Not Detected		
FANCG	Seq	DNA-Tumor	Mutation Not Detected		
FANCI	Seq	DNA-Tumor	Mutation Not Detected		
FANCL	CNA-Seq	DNA-Tumor	Amplification Not Detected		
FANCL	Seq	DNA-Tumor	Mutation Not Detected		
FANCM	Seq	DNA-Tumor	Mutation Not Detected		
FAS	CNA-Seq	DNA-Tumor	Amplification Not Detected		
FAS	Seq	DNA-Tumor	Mutation Not Detected		
FAT1	Seq	DNA-Tumor	Mutation Not Detected		
FAT3	Seq	DNA-Tumor	Mutation Not Detected		
FBXO11	Seq	DNA-Tumor	Mutation Not Detected		
FBXW7	CNA-Seq	DNA-Tumor	Amplification Not Detected		
FBXW7	Seq	DNA-Tumor	Mutation Not Detected		
FCRL4	CNA-Seq	DNA-Tumor	Amplification Not Detected		
FCRL4	Seq	DNA-Tumor	Mutation Not Detected		
FEN1	Seq	DNA-Tumor	Mutation Not Detected		
FGF10	CNA-Seq	DNA-Tumor	Amplification Not Detected		
FGF10	Seq	DNA-Tumor	Mutation Not Detected		
FGF12	Seq	DNA-Tumor	Mutation Not Detected		
FGF14	Seq	DNA-Tumor	Mutation Not Detected		
FGF19	CNA-Seq	DNA-Tumor	Amplification Not Detected		
FGF19	Seq	DNA-Tumor	Mutation Not Detected		
FGF23	CNA-Seq	DNA-Tumor	Amplification Not Detected		
FGF23	Seq	DNA-Tumor	Mutation Not Detected		
FGF3	CNA-Seq	DNA-Tumor	Amplification Not Detected		
FGF3	Seq	DNA-Tumor	Mutation Not Detected		
FGF4	CNA-Seq	DNA-Tumor	Amplification Not Detected		
FGF4	Seq	DNA-Tumor	Mutation Not Detected		
FGF6	Seq	DNA-Tumor	Mutation Not Detected		
FGFR1	CNA-Seq	DNA-Tumor	Amplification Not Detected		
FGFR1	Seq	DNA-Tumor	Mutation Not Detected		
FGFR1	Seq	RNA-Tumor	Fusion Not Detected		
FGFR1OP	CNA-Seq	DNA-Tumor	Amplification Not Detected		
FGFR1OP	Seq	DNA-Tumor	Mutation Not Detected		
FGFR2	CNA-Seq	DNA-Tumor	Amplification Not Detected		
FGFR2	Seq	DNA-Tumor	Mutation Not Detected		
FGFR2	Seq	RNA-Tumor	Fusion Not Detected		
FGFR3	CNA-Seq	DNA-Tumor	Amplification Not Detected		
FGFR3	Seq	DNA-Tumor	Mutation Not Detected		
FGFR3	Seq	RNA-Tumor	Fusion Not Detected		
FGFR4	CNA-Seq	DNA-Tumor	Amplification Not Detected		
FGFR4	Seq	DNA-Tumor	Mutation Not Detected		
FGR	Seq	RNA-Tumor	Fusion Not Detected		
FH	CNA-Seq	DNA-Tumor	Amplification Not Detected		
FH	Seq	DNA-Tumor	Mutation Not Detected		
FHIT	CNA-Seq	DNA-Tumor	Amplification Not Detected		
FHIT	Seq	DNA-Tumor	Mutation Not Detected		
FIP1L1	Seq	DNA-Tumor	Mutation Not Detected		
FLCN	CNA-Seq	DNA-Tumor	Amplification Not Detected		
FLCN	Seq	DNA-Tumor	Mutation Not Detected		
FLI1	CNA-Seq	DNA-Tumor	Amplification Not Detected		
FLI1	Seq	DNA-Tumor	Mutation Not Detected		
FLT1	CNA-Seq	DNA-Tumor	Amplification Not Detected		
FLT1	Seq	DNA-Tumor	Mutation Not Detected		
FLT3	CNA-Seq	DNA-Tumor	Amplification Not Detected		
FLT3	Seq	DNA-Tumor	Mutation Not Detected		
FLT4	CNA-Seq	DNA-Tumor	Amplification Not Detected		
FLT4	Seq	DNA-Tumor	Mutation Not Detected		
FNBP1	CNA-Seq	DNA-Tumor	Amplification Not Detected		
FNBP1	Seq	DNA-Tumor	Mutation Not Detected		
FOXA1	CNA-Seq	DNA-Tumor	Amplification Not Detected		
FOXA1	Seq	DNA-Tumor	Mutation Not Detected		
FOXL2	Seq	DNA-Tumor	Mutation Not Detected		
FOXO1	CNA-Seq	DNA-Tumor	Amplification Not Detected		
FOXO1	Seq	DNA-Tumor	Mutation Not Detected		
FOXO3	Seq	DNA-Tumor	Mutation Not Detected		
FOXO4	Seq	DNA-Tumor	Mutation Not Detected		
FOXP1	CNA-Seq	DNA-Tumor	Amplification Not Detected		
FOXP1	Seq	DNA-Tumor	Mutation Not Detected		
FRS2	Seq	DNA-Tumor	Mutation Not Detected		
FSTL3	Seq	DNA-Tumor	Mutation Not Detected		
FUBP1	CNA-Seq	DNA-Tumor	Amplification Not Detected		
FUBP1	Seq	DNA-Tumor	Mutation Not Detected		
FUS	CNA-Seq	DNA-Tumor	Amplification Not Detected		
FUS	Seq	DNA-Tumor	Mutation Not Detected		
FYN	Seq	DNA-Tumor	Mutation Not Detected		
GABRA6	Seq	DNA-Tumor	Mutation Not Detected		
GALNT12	Seq	DNA-Tumor	Mutation Not Detected		
GATA1	Seq	DNA-Tumor	Mutation Not Detected		
GATA2	Seq	DNA-Tumor	Mutation Not Detected		
GATA3	CNA-Seq	DNA-Tumor	Amplification Not Detected		
GATA3	Seq	DNA-Tumor	Mutation Not Detected		
GATA4	Seq	DNA-Tumor	Mutation Not Detected		
GATA6	Seq	DNA-Tumor	Mutation Not Detected		
GEN1	Seq	DNA-Tumor	Indeterminate		
Genomic LOH	Seq	DNA-Tumor	Low		0
GID4	CNA-Seq	DNA-Tumor	Amplification Not Detected		
GID4	Seq	DNA-Tumor	Mutation Not Detected		
GLI1	Seq	DNA-Tumor	Indeterminate		
GLI2	Seq	DNA-Tumor	Mutation Not Detected		
GMPS	CNA-Seq	DNA-Tumor	Amplification Not Detected		
GMPS	Seq	DNA-Tumor	Unclassified Variant	Exon 9 | p.G367S	58
GNA11	Seq	DNA-Tumor	Mutation Not Detected		
GNA13	CNA-Seq	DNA-Tumor	Amplification Not Detected		
GNA13	Seq	DNA-Tumor	Mutation Not Detected		
GNAQ	CNA-Seq	DNA-Tumor	Amplification Not Detected		
GNAQ	Seq	DNA-Tumor	Mutation Not Detected		
GNAS	CNA-Seq	DNA-Tumor	Amplification Not Detected		
GNAS	Seq	DNA-Tumor	Mutation Not Detected		
GOPC	Seq	DNA-Tumor	Indeterminate		
GPC3	Seq	DNA-Tumor	Mutation Not Detected		
GPS2	Seq	DNA-Tumor	Mutation Not Detected		
GREM1	Seq	DNA-Tumor	Mutation Not Detected		
GRIN2A	CNA-Seq	DNA-Tumor	Amplification Not Detected		
GRIN2A	Seq	DNA-Tumor	Mutation Not Detected		
GRM3	Seq	DNA-Tumor	Mutation Not Detected		
GSK3B	Seq	DNA-Tumor	Mutation Not Detected		
H2AFX	Seq	DNA-Tumor	Mutation Not Detected		
H3F3A	CNA-Seq	DNA-Tumor	Amplification Not Detected		
H3F3A	Seq	DNA-Tumor	Mutation Not Detected		
H3F3B	CNA-Seq	DNA-Tumor	Amplification Not Detected		
H3F3B	Seq	DNA-Tumor	Mutation Not Detected		
HDAC1	Seq	DNA-Tumor	Mutation Not Detected		
HEY1	Seq	DNA-Tumor	Mutation Not Detected		
HGF	Seq	DNA-Tumor	Mutation Not Detected		
HIF1A	Seq	DNA-Tumor	Mutation Not Detected		
HIST1H3B	Seq	DNA-Tumor	Mutation Not Detected		
HIST1H3C	Seq	DNA-Tumor	Mutation Not Detected		
HIST1H4I	Seq	DNA-Tumor	Mutation Not Detected		
HLF	Seq	DNA-Tumor	Mutation Not Detected		
HMGA2	CNA-Seq	DNA-Tumor	Amplification Not Detected		
HMGA2	Seq	DNA-Tumor	Mutation Not Detected		
HMGN2P46	Seq	DNA-Tumor	Indeterminate		
HNF1A	Seq	DNA-Tumor	Mutation Not Detected		
HOOK3	CNA-Seq	DNA-Tumor	Amplification Not Detected		
HOOK3	Seq	DNA-Tumor	Mutation Not Detected		
HOXA11	Seq	DNA-Tumor	Mutation Not Detected		
HOXA13	Seq	DNA-Tumor	Mutation Not Detected		
HOXA9	Seq	DNA-Tumor	Mutation Not Detected		
HOXB13	Seq	DNA-Tumor	Mutation Not Detected		
HOXD13	Seq	DNA-Tumor	Mutation Not Detected		
HRAS	Seq	DNA-Tumor	Mutation Not Detected		
HRG	Seq	DNA-Tumor	Mutation Not Detected		
HSD3B1	Seq	DNA-Tumor	Mutation Not Detected		
HSP90AA1	CNA-Seq	DNA-Tumor	Amplification Not Detected		
HSP90AA1	Seq	DNA-Tumor	Mutation Not Detected		
HSP90AB1	CNA-Seq	DNA-Tumor	Amplification Not Detected		
HSP90AB1	Seq	DNA-Tumor	Mutation Not Detected		
ID2	Seq	DNA-Tumor	Mutation Not Detected		
ID3	Seq	DNA-Tumor	Mutation Not Detected		
IDH1	CNA-Seq	DNA-Tumor	Amplification Not Detected		
IDH1	Seq	DNA-Tumor	Mutation Not Detected		
IDH2	CNA-Seq	DNA-Tumor	Amplification Not Detected		
IDH2	Seq	DNA-Tumor	Mutation Not Detected		
IFNGR1	Seq	DNA-Tumor	Mutation Not Detected		
IGF1R	CNA-Seq	DNA-Tumor	Amplification Not Detected		
IGF1R	Seq	DNA-Tumor	Mutation Not Detected		
IGF2	Seq	DNA-Tumor	Mutation Not Detected		
IKBKE	Seq	DNA-Tumor	Unclassified Variant	Exon 10 | p.S377N	45
IKZF1	CNA-Seq	DNA-Tumor	Amplification Not Detected		
IKZF1	Seq	DNA-Tumor	Mutation Not Detected		
IL7R	CNA-Seq	DNA-Tumor	Amplification Not Detected		
IL7R	Seq	DNA-Tumor	Mutation Not Detected		
INHBA	Seq	DNA-Tumor	Mutation Not Detected		
INPP4B	Seq	DNA-Tumor	Mutation Not Detected		
INSR	Seq	RNA-Tumor	Fusion Not Detected		
IRF1	Seq	DNA-Tumor	Mutation Not Detected		
IRF2	Seq	DNA-Tumor	Mutation Not Detected		
IRF4	CNA-Seq	DNA-Tumor	Amplification Not Detected		
IRF4	Seq	DNA-Tumor	Mutation Not Detected		
IRS2	Seq	DNA-Tumor	Mutation Not Detected		
ITK	CNA-Seq	DNA-Tumor	Amplification Not Detected		
ITK	Seq	DNA-Tumor	Mutation Not Detected		
JAK1	CNA-Seq	DNA-Tumor	Amplification Not Detected		
JAK1	Seq	DNA-Tumor	Mutation Not Detected		
JAK2	CNA-Seq	DNA-Tumor	Amplification Not Detected		
JAK2	Seq	DNA-Tumor	Mutation Not Detected		
JAK3	CNA-Seq	DNA-Tumor	Amplification Not Detected		
JAK3	Seq	DNA-Tumor	Mutation Not Detected		
JAZF1	CNA-Seq	DNA-Tumor	Amplification Not Detected		
JAZF1	Seq	DNA-Tumor	Mutation Not Detected		
JUN	Seq	DNA-Tumor	Mutation Not Detected		
KAT6A	Seq	DNA-Tumor	Mutation Not Detected		
KAT6B	Seq	DNA-Tumor	Mutation Not Detected		
KCNJ5	Seq	DNA-Tumor	Mutation Not Detected		
KDM5A	Seq	DNA-Tumor	Mutation Not Detected		
KDM5C	Seq	DNA-Tumor	Mutation Not Detected		
KDM6A	Seq	DNA-Tumor	Mutation Not Detected		
KDR (VEGFR2)	CNA-Seq	DNA-Tumor	Amplification Not Detected		
KDR (VEGFR2)	Seq	DNA-Tumor	Mutation Not Detected		
KDSR	Seq	DNA-Tumor	Mutation Not Detected		
KEAP1	CNA-Seq	DNA-Tumor	Amplification Not Detected		
KEAP1	Seq	DNA-Tumor	Mutation Not Detected		
KEL	Seq	DNA-Tumor	Mutation Not Detected		
KIAA1549	CNA-Seq	DNA-Tumor	Amplification Not Detected		
KIAA1549	Seq	DNA-Tumor	Indeterminate		
KIF1B	Seq	DNA-Tumor	Mutation Not Detected		
KIF5B	CNA-Seq	DNA-Tumor	Amplification Not Detected		
KIF5B	Seq	DNA-Tumor	Mutation Not Detected		
KIT	CNA-Seq	DNA-Tumor	Amplification Not Detected		
KIT	Seq	DNA-Tumor	Mutation Not Detected		
KLF4	Seq	DNA-Tumor	Mutation Not Detected		
KLHL6	CNA-Seq	DNA-Tumor	Amplification Not Detected		
KLHL6	Seq	DNA-Tumor	Mutation Not Detected		
KLK2	Seq	DNA-Tumor	Mutation Not Detected		
KMT2A	CNA-Seq	DNA-Tumor	Amplification Not Detected		
KMT2A	Seq	DNA-Tumor	Mutation Not Detected		
KMT2C	CNA-Seq	DNA-Tumor	Amplification Not Detected		
KMT2C	Seq	DNA-Tumor	Mutation Not Detected		
KMT2D	CNA-Seq	DNA-Tumor	Amplification Not Detected		
KMT2D	Seq	DNA-Tumor	Mutation Not Detected		
KRAS	CNA-Seq	DNA-Tumor	Amplification Not Detected		
KRAS	Seq	DNA-Tumor	Pathogenic Variant	Exon 2 | p.G13C	35
LCK	CNA-Seq	DNA-Tumor	Amplification Not Detected		
LCK	Seq	DNA-Tumor	Mutation Not Detected		
LDLR	Seq	DNA-Tumor	Mutation Not Detected		
LHFPL6	CNA-Seq	DNA-Tumor	Amplification Not Detected		
LHFPL6	Seq	DNA-Tumor	Mutation Not Detected		
LIFR	CNA-Seq	DNA-Tumor	Amplification Not Detected		
LIFR	Seq	DNA-Tumor	Indeterminate		
LIG1	Seq	DNA-Tumor	Mutation Not Detected		
LMNA	Seq	DNA-Tumor	Mutation Not Detected		
LMO1	Seq	DNA-Tumor	Mutation Not Detected		
LMO2	Seq	DNA-Tumor	Indeterminate		
LPP	CNA-Seq	DNA-Tumor	Amplification Not Detected		
LPP	Seq	DNA-Tumor	Mutation Not Detected		
LRP1B	CNA-Seq	DNA-Tumor	Amplification Not Detected		
LRP1B	Seq	DNA-Tumor	Mutation Not Detected		
LTK	Seq	DNA-Tumor	Indeterminate		
LYN	Seq	DNA-Tumor	Mutation Not Detected		
LZTR1	Seq	DNA-Tumor	Mutation Not Detected		
MAF	CNA-Seq	DNA-Tumor	Amplification Not Detected		
MAF	Seq	DNA-Tumor	Mutation Not Detected		
MAFB	Seq	DNA-Tumor	Mutation Not Detected		
MAGI2	Seq	DNA-Tumor	Indeterminate		
MALT1	Seq	DNA-Tumor	Indeterminate		
MAML2	CNA-Seq	DNA-Tumor	Amplification Not Detected		
MAML2	Seq	DNA-Tumor	Mutation Not Detected		
MAML2	Seq	RNA-Tumor	Fusion Not Detected		
MAP2K1 (MEK1)	CNA-Seq	DNA-Tumor	Amplification Not Detected		
MAP2K1 (MEK1)	Seq	DNA-Tumor	Mutation Not Detected		
MAP2K2 (MEK2)	CNA-Seq	DNA-Tumor	Amplification Not Detected		
MAP2K2 (MEK2)	Seq	DNA-Tumor	Mutation Not Detected		
MAP2K4	CNA-Seq	DNA-Tumor	Amplification Not Detected		
MAP2K4	Seq	DNA-Tumor	Mutation Not Detected		
MAP3K1	CNA-Seq	DNA-Tumor	Amplification Not Detected		
MAP3K1	Seq	DNA-Tumor	Mutation Not Detected		
MAP3K13	Seq	DNA-Tumor	Mutation Not Detected		
MAPK1	Seq	DNA-Tumor	Mutation Not Detected		
MAPK3	Seq	DNA-Tumor	Mutation Not Detected		
MAST1	Seq	RNA-Tumor	Fusion Not Detected		
MAST2	Seq	RNA-Tumor	Fusion Not Detected		
MAX	Seq	DNA-Tumor	Mutation Not Detected		
MBD4	Seq	DNA-Tumor	Mutation Not Detected		
MCL1	CNA-Seq	DNA-Tumor	Amplification Not Detected		
MCL1	Seq	DNA-Tumor	Mutation Not Detected		
MDC1	Seq	DNA-Tumor	Mutation Not Detected		
MDM2	CNA-Seq	DNA-Tumor	Amplification Not Detected		
MDM2	Seq	DNA-Tumor	Mutation Not Detected		
MDM4	CNA-Seq	DNA-Tumor	Amplification Not Detected		
MDM4	Seq	DNA-Tumor	Mutation Not Detected		
MDS2	CNA-Seq	DNA-Tumor	Amplification Not Detected		
MDS2	Seq	DNA-Tumor	Mutation Not Detected		
MECOM	Seq	DNA-Tumor	Mutation Not Detected		
MED12	Seq	DNA-Tumor	Indeterminate		
MEF2B	CNA-Seq	DNA-Tumor	Amplification Not Detected		
MEF2B	Seq	DNA-Tumor	Mutation Not Detected		
MEN1	CNA-Seq	DNA-Tumor	Amplification Not Detected		
MEN1	Seq	DNA-Tumor	Mutation Not Detected		
MERTK	Seq	DNA-Tumor	Mutation Not Detected		
MET	CNA-Seq	DNA-Tumor	Amplification Not Detected		
MET	Seq	DNA-Tumor	Mutation Not Detected		
MET	Seq	RNA-Tumor	Fusion Not Detected		
MET	Seq	RNA-Tumor	Variant Transcript Not Detected		
MGA	Seq	DNA-Tumor	Mutation Not Detected		
MGMT	Seq	DNA-Tumor	Mutation Not Detected		
Mismatch Repair Status	IHC	Protein	Proficient		
MITF	CNA-Seq	DNA-Tumor	Amplification Not Detected		
MITF	Seq	DNA-Tumor	Mutation Not Detected		
MKNK1	Seq	DNA-Tumor	Indeterminate		
MLF1	CNA-Seq	DNA-Tumor	Amplification Not Detected		
MLF1	Seq	DNA-Tumor	Mutation Not Detected		
MLH1	CNA-Seq	DNA-Tumor	Amplification Not Detected		
MLH1	IHC	Protein	Positive | 1+, 80%		
MLH1	Seq	DNA-Tumor	Mutation Not Detected		
MLH3	Seq	DNA-Tumor	Unclassified Variant	Exon 2 | p.Q563R	48
MLLT10	CNA-Seq	DNA-Tumor	Amplification Not Detected		
MLLT10	Seq	DNA-Tumor	Mutation Not Detected		
MLLT11	Seq	DNA-Tumor	Mutation Not Detected		
MLLT3	CNA-Seq	DNA-Tumor	Amplification Not Detected		
MLLT3	Seq	DNA-Tumor	Mutation Not Detected		
MN1	Seq	DNA-Tumor	Mutation Not Detected		
MPL	Seq	DNA-Tumor	Mutation Not Detected		
MRE11	CNA-Seq	DNA-Tumor	Amplification Not Detected		
MRE11	Seq	DNA-Tumor	Mutation Not Detected		
MSH2	CNA-Seq	DNA-Tumor	Amplification Not Detected		
MSH2	IHC	Protein	Positive | 2+, 95%		
MSH2	Seq	DNA-Tumor	Mutation Not Detected		
MSH3	Seq	DNA-Tumor	Mutation Not Detected		
MSH6	CNA-Seq	DNA-Tumor	Amplification Not Detected		
MSH6	IHC	Protein	Positive | 2+, 95%		
MSH6	Seq	DNA-Tumor	Mutation Not Detected		
MSI	Seq	DNA-Tumor	Stable		0
MSI2	CNA-Seq	DNA-Tumor	Amplification Not Detected		
MSI2	Seq	DNA-Tumor	Mutation Not Detected		
MSMB	Seq	RNA-Tumor	Fusion Not Detected		
MST1R (RON)	Seq	DNA-Tumor	Mutation Not Detected		
MTAP	Seq	DNA-Tumor	Mutation Not Detected		
MTCP1	Seq	DNA-Tumor	Mutation Not Detected		
MTOR	CNA-Seq	DNA-Tumor	Amplification Not Detected		
MTOR	Seq	DNA-Tumor	Mutation Not Detected		
MUC1	Seq	DNA-Tumor	Mutation Not Detected		
MUS81	Seq	DNA-Tumor	Mutation Not Detected		
MUSK	Seq	RNA-Tumor	Fusion Not Detected		
MUTYH	Seq	DNA-Tumor	Mutation Not Detected		
MYB	CNA-Seq	DNA-Tumor	Amplification Not Detected		
MYB	Seq	DNA-Tumor	Indeterminate		
MYB	Seq	RNA-Tumor	Fusion Not Detected		
MYC	CNA-Seq	DNA-Tumor	Amplification Not Detected		
MYC	Seq	DNA-Tumor	Mutation Not Detected		
MYCL	Seq	DNA-Tumor	Mutation Not Detected		
MYCN	CNA-Seq	DNA-Tumor	Amplification Not Detected		
MYCN	Seq	DNA-Tumor	Mutation Not Detected		
MYD88	CNA-Seq	DNA-Tumor	Amplification Not Detected		
MYD88	Seq	DNA-Tumor	Mutation Not Detected		
MYH11	Seq	DNA-Tumor	Mutation Not Detected		
MYH9	Seq	DNA-Tumor	Mutation Not Detected		
NBN	Seq	DNA-Tumor	Mutation Not Detected		
NCOA2	CNA-Seq	DNA-Tumor	Amplification Not Detected		
NCOA2	Seq	DNA-Tumor	Mutation Not Detected		
NCOA3	Seq	DNA-Tumor	Mutation Not Detected		
NCOA4	Seq	DNA-Tumor	Mutation Not Detected		
NCOR1	Seq	DNA-Tumor	Mutation Not Detected		
NDRG1	Seq	DNA-Tumor	Mutation Not Detected		
NF1	CNA-Seq	DNA-Tumor	Amplification Not Detected		
NF1	Seq	DNA-Tumor	Mutation Not Detected		
NF2	CNA-Seq	DNA-Tumor	Amplification Not Detected		
NF2	Seq	DNA-Tumor	Mutation Not Detected		
NFE2L2	CNA-Seq	DNA-Tumor	Amplification Not Detected		
NFE2L2	Seq	DNA-Tumor	Mutation Not Detected		
NFIB	CNA-Seq	DNA-Tumor	Amplification Not Detected		
NFIB	Seq	DNA-Tumor	Mutation Not Detected		
NFKB2	CNA-Seq	DNA-Tumor	Amplification Not Detected		
NFKB2	Seq	DNA-Tumor	Mutation Not Detected		
NFKBIA	CNA-Seq	DNA-Tumor	Amplification Not Detected		
NFKBIA	Seq	DNA-Tumor	Mutation Not Detected		
NFKBIE	Seq	DNA-Tumor	Mutation Not Detected		
NIN	CNA-Seq	DNA-Tumor	Amplification Not Detected		
NIN	Seq	DNA-Tumor	Mutation Not Detected		
NKX2-1	Seq	DNA-Tumor	Mutation Not Detected		
NONO	Seq	DNA-Tumor	Mutation Not Detected		
NOTCH1	Seq	DNA-Tumor	Mutation Not Detected		
NOTCH1	Seq	RNA-Tumor	Fusion Not Detected		
NOTCH2	CNA-Seq	DNA-Tumor	Amplification Not Detected		
NOTCH2	Seq	DNA-Tumor	Mutation Not Detected		
NOTCH2	Seq	RNA-Tumor	Fusion Not Detected		
NOTCH3	Seq	DNA-Tumor	Indeterminate		
NPM1	CNA-Seq	DNA-Tumor	Amplification Not Detected		
NPM1	Seq	DNA-Tumor	Indeterminate		
NR4A3	CNA-Seq	DNA-Tumor	Amplification Not Detected		
NR4A3	Seq	DNA-Tumor	Mutation Not Detected		
NRAS	Seq	DNA-Tumor	Mutation Not Detected		
NRG1	Seq	RNA-Tumor	Fusion Not Detected		
NSD1	CNA-Seq	DNA-Tumor	Amplification Not Detected		
NSD1	Seq	DNA-Tumor	Mutation Not Detected		
NSD2	Seq	DNA-Tumor	Mutation Not Detected		
NSD3	Seq	DNA-Tumor	Mutation Not Detected		
NT5C2	Seq	DNA-Tumor	Mutation Not Detected		
NTHL1	Seq	DNA-Tumor	Mutation Not Detected		
NTRK1	CNA-Seq	DNA-Tumor	Amplification Not Detected		
NTRK1	Seq	DNA-Tumor	Mutation Not Detected		
NTRK1	Seq	RNA-Tumor	Fusion Not Detected		
NTRK2	CNA-Seq	DNA-Tumor	Amplification Not Detected		
NTRK2	Seq	DNA-Tumor	Mutation Not Detected		
NTRK2	Seq	RNA-Tumor	Fusion Not Detected		
NTRK3	CNA-Seq	DNA-Tumor	Amplification Not Detected		
NTRK3	Seq	DNA-Tumor	Mutation Not Detected		
NTRK3	Seq	RNA-Tumor	Fusion Not Detected		
NUMBL	Seq	RNA-Tumor	Fusion Not Detected		
NUP214	CNA-Seq	DNA-Tumor	Amplification Not Detected		
NUP214	Seq	DNA-Tumor	Mutation Not Detected		
NUP93	CNA-Seq	DNA-Tumor	Amplification Not Detected		
NUP93	Seq	DNA-Tumor	Mutation Not Detected		
NUP98	CNA-Seq	DNA-Tumor	Amplification Not Detected		
NUP98	Seq	DNA-Tumor	Mutation Not Detected		
NUTM1	CNA-Seq	DNA-Tumor	Amplification Not Detected		
NUTM1	Seq	DNA-Tumor	Mutation Not Detected		
NUTM1	Seq	RNA-Tumor	Fusion Not Detected		
OLIG2	Seq	DNA-Tumor	Mutation Not Detected		
P2RY8	Seq	DNA-Tumor	Mutation Not Detected		
PAFAH1B2	Seq	DNA-Tumor	Mutation Not Detected		
PAK1	Seq	DNA-Tumor	Mutation Not Detected		
PAK3	Seq	DNA-Tumor	Mutation Not Detected		
PALB2	CNA-Seq	DNA-Tumor	Amplification Not Detected		
PALB2	Seq	DNA-Tumor	Mutation Not Detected		
PARP1	Seq	DNA-Tumor	Mutation Not Detected		
PARP2	Seq	DNA-Tumor	Mutation Not Detected		
PARP3	Seq	DNA-Tumor	Mutation Not Detected		
PAX3	CNA-Seq	DNA-Tumor	Amplification Not Detected		
PAX3	Seq	DNA-Tumor	Mutation Not Detected		
PAX5	CNA-Seq	DNA-Tumor	Amplification Not Detected		
PAX5	Seq	DNA-Tumor	Mutation Not Detected		
PAX7	Seq	DNA-Tumor	Mutation Not Detected		
PAX8	Seq	DNA-Tumor	Mutation Not Detected		
PBRM1	CNA-Seq	DNA-Tumor	Amplification Not Detected		
PBRM1	Seq	DNA-Tumor	Mutation Not Detected		
PBX1	CNA-Seq	DNA-Tumor	Amplification Not Detected		
PBX1	Seq	DNA-Tumor	Mutation Not Detected		
PCM1	CNA-Seq	DNA-Tumor	Amplification Not Detected		
PCM1	Seq	DNA-Tumor	Mutation Not Detected		
PDCD1	CNA-Seq	DNA-Tumor	Amplification Not Detected		
PDCD1	Seq	DNA-Tumor	Mutation Not Detected		
PDCD1LG2	CNA-Seq	DNA-Tumor	Amplification Not Detected		
PDCD1LG2	Seq	DNA-Tumor	Mutation Not Detected		
PDE4DIP	Seq	DNA-Tumor	Mutation Not Detected		
PDGFB	Seq	DNA-Tumor	Mutation Not Detected		
PDGFRA	CNA-Seq	DNA-Tumor	Amplification Not Detected		
PDGFRA	Seq	DNA-Tumor	Mutation Not Detected		
PDGFRA	Seq	RNA-Tumor	Fusion Not Detected		
PDGFRB	CNA-Seq	DNA-Tumor	Amplification Not Detected		
PDGFRB	Seq	DNA-Tumor	Mutation Not Detected		
PDGFRB	Seq	RNA-Tumor	Fusion Not Detected		
PDK1	Seq	DNA-Tumor	Mutation Not Detected		
PD-L1 (SP142)	IHC	Protein	Negative | 0, 0%		
PDZD8	Seq	RNA-Tumor	Unclassified Fusion Detected	PDZD8:EFCAB5 |	
PER1	CNA-Seq	DNA-Tumor	Amplification Not Detected		
PER1	Seq	DNA-Tumor	Mutation Not Detected		
PGAP3	Seq	RNA-Tumor	Unclassified Fusion Detected	PGAP3:IKZF3 |	
PGAP3	Seq	RNA-Tumor	Unclassified Fusion Detected	PGAP3:IKZF3 |	
PHF6	Seq	DNA-Tumor	Mutation Not Detected		
PHOX2B	Seq	DNA-Tumor	Mutation Not Detected		
PIK3C2B	Seq	DNA-Tumor	Mutation Not Detected		
PIK3C2G	Seq	DNA-Tumor	Indeterminate		
PIK3CA	CNA-Seq	DNA-Tumor	Amplification Not Detected		
PIK3CA	Seq	DNA-Tumor	Pathogenic Variant	Exon 2 | p.R88Q	45
PIK3CA	Seq	RNA-Tumor	Fusion Not Detected		
PIK3CB	Seq	DNA-Tumor	Indeterminate		
PIK3CD	Seq	DNA-Tumor	Mutation Not Detected		
PIK3CG	Seq	DNA-Tumor	Mutation Not Detected		
PIK3R1	CNA-Seq	DNA-Tumor	Amplification Not Detected		
PIK3R1	Seq	DNA-Tumor	Mutation Not Detected		
PIK3R2	Seq	DNA-Tumor	Indeterminate		
PIM1	CNA-Seq	DNA-Tumor	Amplification Not Detected		
PIM1	Seq	DNA-Tumor	Mutation Not Detected		
PITPNC1	Seq	RNA-Tumor	Unclassified Fusion Detected	PITPNC1:CACNG4 |	
PKN1	Seq	RNA-Tumor	Fusion Not Detected		
PLAG1	Seq	DNA-Tumor	Mutation Not Detected		
PLCG2	Seq	DNA-Tumor	Mutation Not Detected		
PML	Seq	DNA-Tumor	Mutation Not Detected		
PMS1	Seq	DNA-Tumor	Mutation Not Detected		
PMS2	CNA-Seq	DNA-Tumor	Amplification Not Detected		
PMS2	IHC	Protein	Positive | 1+, 70%		
PMS2	Seq	DNA-Tumor	Mutation Not Detected		
POLD1	Seq	DNA-Tumor	Mutation Not Detected		
POLD2	Seq	DNA-Tumor	Mutation Not Detected		
POLD3	Seq	DNA-Tumor	Mutation Not Detected		
POLD4	Seq	DNA-Tumor	Mutation Not Detected		
POLE	CNA-Seq	DNA-Tumor	Amplification Not Detected		
POLE	Seq	DNA-Tumor	Mutation Not Detected		
POLH	Seq	DNA-Tumor	Unclassified Variant	Exon 11 | p.E495K	51
POLH	Seq	DNA-Tumor	Unclassified Variant	Exon 4 | p.G153D	48
POLQ	Seq	DNA-Tumor	Mutation Not Detected		
POT1	CNA-Seq	DNA-Tumor	Amplification Not Detected		
POT1	Seq	DNA-Tumor	Mutation Not Detected		
POU2AF1	CNA-Seq	DNA-Tumor	Amplification Not Detected		
POU2AF1	Seq	DNA-Tumor	Mutation Not Detected		
PPARG	CNA-Seq	DNA-Tumor	Amplification Not Detected		
PPARG	Seq	DNA-Tumor	Mutation Not Detected		
PPARG	Seq	RNA-Tumor	Fusion Not Detected		
PPM1D	Seq	DNA-Tumor	Mutation Not Detected		
PPP2R1A	Seq	DNA-Tumor	Mutation Not Detected		
PPP2R2A	Seq	DNA-Tumor	Mutation Not Detected		
PPP6C	Seq	DNA-Tumor	Mutation Not Detected		
PR	IHC	Protein	Negative | 0, 100%		
PRCC	CNA-Seq	DNA-Tumor	Amplification Not Detected		
PRCC	Seq	DNA-Tumor	Mutation Not Detected		
PRDM1	CNA-Seq	DNA-Tumor	Amplification Not Detected		
PRDM1	Seq	DNA-Tumor	Mutation Not Detected		
PREX2	Seq	DNA-Tumor	Mutation Not Detected		
PRF1	Seq	DNA-Tumor	Mutation Not Detected		
PRKACA	Seq	DNA-Tumor	Indeterminate		
PRKAR1A	CNA-Seq	DNA-Tumor	Amplification Not Detected		
PRKAR1A	Seq	DNA-Tumor	Mutation Not Detected		
PRKCA	Seq	RNA-Tumor	Fusion Not Detected		
PRKCB	Seq	RNA-Tumor	Fusion Not Detected		
PRKCH	Seq	DNA-Tumor	Mutation Not Detected		
PRKCI	Seq	DNA-Tumor	Mutation Not Detected		
PRKDC	Seq	DNA-Tumor	Mutation Not Detected		
PRKN	Seq	DNA-Tumor	Mutation Not Detected		
PRRX1	CNA-Seq	DNA-Tumor	Amplification Not Detected		
PRRX1	Seq	DNA-Tumor	Mutation Not Detected		
PRSS8	Seq	DNA-Tumor	Mutation Not Detected		
PTCH1	CNA-Seq	DNA-Tumor	Amplification Not Detected		
PTCH1	Seq	DNA-Tumor	Mutation Not Detected		
PTCH2	Seq	DNA-Tumor	Mutation Not Detected		
PTEN	CNA-Seq	DNA-Tumor	Amplification Not Detected		
PTEN	IHC	Protein	Positive | 2+, 90%		
PTEN	Seq	DNA-Tumor	Mutation Not Detected		
PTK2B	Seq	DNA-Tumor	Indeterminate		
PTPN11	CNA-Seq	DNA-Tumor	Amplification Not Detected		
PTPN11	Seq	DNA-Tumor	Indeterminate		
PTPN22	Seq	DNA-Tumor	Indeterminate		
PTPRC	CNA-Seq	DNA-Tumor	Amplification Not Detected		
PTPRC	Seq	DNA-Tumor	Mutation Not Detected		
PTPRD	Seq	DNA-Tumor	Indeterminate		
PTPRO	Seq	DNA-Tumor	Indeterminate		
PTPRT	Seq	DNA-Tumor	Indeterminate		
QKI	Seq	DNA-Tumor	Mutation Not Detected		
RABL3	Seq	DNA-Tumor	Mutation Not Detected		
RAC1	CNA-Seq	DNA-Tumor	Amplification Not Detected		
RAC1	Seq	DNA-Tumor	Indeterminate		
RAD21	Seq	DNA-Tumor	Mutation Not Detected		
RAD50	CNA-Seq	DNA-Tumor	Amplification Not Detected		
RAD50	Seq	DNA-Tumor	Mutation Not Detected		
RAD51	Seq	DNA-Tumor	Mutation Not Detected		
RAD51B	Seq	DNA-Tumor	Mutation Not Detected		
RAD51C	Seq	DNA-Tumor	Mutation Not Detected		
RAD51D	Seq	DNA-Tumor	Mutation Not Detected		
RAD52	Seq	DNA-Tumor	Mutation Not Detected		
RAD54B	Seq	DNA-Tumor	Mutation Not Detected		
RAD54L	Seq	DNA-Tumor	Mutation Not Detected		
RAF1	CNA-Seq	DNA-Tumor	Amplification Not Detected		
RAF1	Seq	DNA-Tumor	Mutation Not Detected		
RAF1	Seq	RNA-Tumor	Fusion Not Detected		
RANBP2	Seq	DNA-Tumor	Mutation Not Detected		
RARA	Seq	DNA-Tumor	Mutation Not Detected		
RASA1	Seq	DNA-Tumor	Indeterminate		
RB1	CNA-Seq	DNA-Tumor	Amplification Not Detected		
RB1	Seq	DNA-Tumor	Indeterminate		
RBBP8	Seq	DNA-Tumor	Mutation Not Detected		
RBM10	Seq	DNA-Tumor	Mutation Not Detected		
RCAN1	Seq	DNA-Tumor	Mutation Not Detected		
RECQL4	Seq	DNA-Tumor	Mutation Not Detected		
REL	Seq	DNA-Tumor	Mutation Not Detected		
RELA	Seq	DNA-Tumor	Indeterminate		
RELA	Seq	RNA-Tumor	Fusion Not Detected		
RET	CNA-Seq	DNA-Tumor	Amplification Not Detected		
RET	Seq	DNA-Tumor	Mutation Not Detected		
RET	Seq	RNA-Tumor	Fusion Not Detected		
RHEB	Seq	DNA-Tumor	Mutation Not Detected		
RHOA	Seq	DNA-Tumor	Mutation Not Detected		
RHOH	Seq	DNA-Tumor	Mutation Not Detected		
RICTOR	CNA-Seq	DNA-Tumor	Amplification Not Detected		
RICTOR	Seq	DNA-Tumor	Mutation Not Detected		
RINT1	Seq	DNA-Tumor	Mutation Not Detected		
RIT1	Seq	DNA-Tumor	Mutation Not Detected		
RMI2	Seq	DNA-Tumor	Mutation Not Detected		
RNF43	CNA-Seq	DNA-Tumor	Amplification Not Detected		
RNF43	Seq	DNA-Tumor	Mutation Not Detected		
ROS1	CNA-Seq	DNA-Tumor	Amplification Not Detected		
ROS1	Seq	DNA-Tumor	Mutation Not Detected		
ROS1	Seq	RNA-Tumor	Fusion Not Detected		
RPA1	Seq	DNA-Tumor	Indeterminate		
RPA2	Seq	DNA-Tumor	Mutation Not Detected		
RPA3	Seq	DNA-Tumor	Mutation Not Detected		
RPA4	Seq	DNA-Tumor	Mutation Not Detected		
RPL10	Seq	DNA-Tumor	Mutation Not Detected		
RPL22	CNA-Seq	DNA-Tumor	Amplification Not Detected		
RPL22	Seq	DNA-Tumor	Mutation Not Detected		
RPL5	Seq	DNA-Tumor	Mutation Not Detected		
RPN1	CNA-Seq	DNA-Tumor	Amplification Not Detected		
RPN1	Seq	DNA-Tumor	Mutation Not Detected		
RPTOR	Seq	DNA-Tumor	Mutation Not Detected		
RRAS2	Seq	DNA-Tumor	Mutation Not Detected		
RSPO1	Seq	DNA-Tumor	Mutation Not Detected		
RSPO2	Seq	DNA-Tumor	Mutation Not Detected		
RSPO2	Seq	RNA-Tumor	Fusion Not Detected		
RSPO3	Seq	DNA-Tumor	Mutation Not Detected		
RSPO3	Seq	RNA-Tumor	Fusion Not Detected		
RUNX1	CNA-Seq	DNA-Tumor	Amplification Not Detected		
RUNX1	Seq	DNA-Tumor	Mutation Not Detected		
RUNx1T1	CNA-Seq	DNA-Tumor	Amplification Not Detected		
RUNx1T1	Seq	DNA-Tumor	Mutation Not Detected		
SBDS	CNA-Seq	DNA-Tumor	Amplification Not Detected		
SBDS	Seq	DNA-Tumor	Mutation Not Detected		
SDC4	CNA-Seq	DNA-Tumor	Amplification Not Detected		
SDC4	Seq	DNA-Tumor	Mutation Not Detected		
SDHA	Seq	DNA-Tumor	Mutation Not Detected		
SDHAF2	CNA-Seq	DNA-Tumor	Amplification Not Detected		
SDHAF2	Seq	DNA-Tumor	Mutation Not Detected		
SDHB	CNA-Seq	DNA-Tumor	Amplification Not Detected		
SDHB	Seq	DNA-Tumor	Mutation Not Detected		
SDHC	CNA-Seq	DNA-Tumor	Amplification Not Detected		
SDHC	Seq	DNA-Tumor	Mutation Not Detected		
SDHD	CNA-Seq	DNA-Tumor	Amplification Not Detected		
SDHD	Seq	DNA-Tumor	Mutation Not Detected		
SEM1	Seq	DNA-Tumor	Mutation Not Detected		
SERPINB3	Seq	DNA-Tumor	Mutation Not Detected		
SET	Seq	DNA-Tumor	Indeterminate		
SETBP1	CNA-Seq	DNA-Tumor	Amplification Not Detected		
SETBP1	Seq	DNA-Tumor	Mutation Not Detected		
SETD2	CNA-Seq	DNA-Tumor	Amplification Not Detected		
SETD2	Seq	DNA-Tumor	Mutation Not Detected		
SF3B1	CNA-Seq	DNA-Tumor	Amplification Not Detected		
SF3B1	Seq	DNA-Tumor	Mutation Not Detected		
SFPQ	Seq	DNA-Tumor	Mutation Not Detected		
SGK1	Seq	DNA-Tumor	Mutation Not Detected		
SH2B3	Seq	DNA-Tumor	Mutation Not Detected		
SLC34A2	CNA-Seq	DNA-Tumor	Amplification Not Detected		
SLC34A2	Seq	DNA-Tumor	Mutation Not Detected		
SLIT2	Seq	DNA-Tumor	Mutation Not Detected		
SLX4	Seq	DNA-Tumor	Mutation Not Detected		
SMAD2	CNA-Seq	DNA-Tumor	Amplification Not Detected		
SMAD2	Seq	DNA-Tumor	Mutation Not Detected		
SMAD3	Seq	DNA-Tumor	Mutation Not Detected		
SMAD4	CNA-Seq	DNA-Tumor	Amplification Not Detected		
SMAD4	Seq	DNA-Tumor	Mutation Not Detected		
SMARCA1	Seq	DNA-Tumor	Indeterminate		
SMARCA4	Seq	DNA-Tumor	Mutation Not Detected		
SMARCB1	CNA-Seq	DNA-Tumor	Amplification Not Detected		
SMARCB1	Seq	DNA-Tumor	Mutation Not Detected		
SMARCE1	CNA-Seq	DNA-Tumor	Amplification Not Detected		
SMARCE1	Seq	DNA-Tumor	Mutation Not Detected		
SMC3	Seq	DNA-Tumor	Mutation Not Detected		
SMO	CNA-Seq	DNA-Tumor	Amplification Not Detected		
SMO	Seq	DNA-Tumor	Mutation Not Detected		
SNCAIP	Seq	DNA-Tumor	Indeterminate		
SNORA50A	Seq	RNA-Tumor	Unclassified Fusion Detected	SNORA50A:CNOT1 |	
SNX29	CNA-Seq	DNA-Tumor	Amplification Not Detected		
SNX29	Seq	DNA-Tumor	Mutation Not Detected		
SOCS1	Seq	DNA-Tumor	Mutation Not Detected		
SOS1	Seq	DNA-Tumor	Mutation Not Detected		
SOX10	CNA-Seq	DNA-Tumor	Amplification Not Detected		
SOX10	Seq	DNA-Tumor	Mutation Not Detected		
SOX2	Seq	DNA-Tumor	Mutation Not Detected		
SOX9	Seq	DNA-Tumor	Mutation Not Detected		
SPECC1	CNA-Seq	DNA-Tumor	Amplification Not Detected		
SPECC1	Seq	DNA-Tumor	Mutation Not Detected		
SPEN	CNA-Seq	DNA-Tumor	Amplification Not Detected		
SPEN	Seq	DNA-Tumor	Mutation Not Detected		
SPOP	Seq	DNA-Tumor	Mutation Not Detected		
SPRED1	Seq	DNA-Tumor	Mutation Not Detected		
SPTA1	Seq	DNA-Tumor	Mutation Not Detected		
SRC	Seq	DNA-Tumor	Mutation Not Detected		
SRGAP3	CNA-Seq	DNA-Tumor	Amplification Not Detected		
SRGAP3	Seq	DNA-Tumor	Mutation Not Detected		
SRSF2	CNA-Seq	DNA-Tumor	Amplification Not Detected		
SRSF2	Seq	DNA-Tumor	Mutation Not Detected		
SRSF3	CNA-Seq	DNA-Tumor	Amplification Not Detected		
SRSF3	Seq	DNA-Tumor	Mutation Not Detected		
SS18	Seq	DNA-Tumor	Mutation Not Detected		
SSBP1	Seq	DNA-Tumor	Mutation Not Detected		
STAG2	Seq	DNA-Tumor	Indeterminate		
STAT3	CNA-Seq	DNA-Tumor	Amplification Not Detected		
STAT3	Seq	DNA-Tumor	Mutation Not Detected		
STAT4	Seq	DNA-Tumor	Mutation Not Detected		
STAT5B	CNA-Seq	DNA-Tumor	Amplification Not Detected		
STAT5B	Seq	DNA-Tumor	Mutation Not Detected		
STAT6	Seq	DNA-Tumor	Mutation Not Detected		
STIL	CNA-Seq	DNA-Tumor	Amplification Not Detected		
STIL	Seq	DNA-Tumor	Mutation Not Detected		
STK11	CNA-Seq	DNA-Tumor	Amplification Not Detected		
STK11	Seq	DNA-Tumor	Mutation Not Detected		
SUFU	CNA-Seq	DNA-Tumor	Amplification Not Detected		
SUFU	Seq	DNA-Tumor	Mutation Not Detected		
SUZ12	CNA-Seq	DNA-Tumor	Amplification Not Detected		
SUZ12	Seq	DNA-Tumor	Mutation Not Detected		
SYK	CNA-Seq	DNA-Tumor	Amplification Not Detected		
SYK	Seq	DNA-Tumor	Mutation Not Detected		
TAF1	Seq	DNA-Tumor	Mutation Not Detected		
TAF15	CNA-Seq	DNA-Tumor	Amplification Not Detected		
TAF15	Seq	DNA-Tumor	Mutation Not Detected		
TAL1	Seq	DNA-Tumor	Mutation Not Detected		
TAL2	Seq	DNA-Tumor	Mutation Not Detected		
TBX3	Seq	DNA-Tumor	Mutation Not Detected		
TBXT	Seq	DNA-Tumor	Mutation Not Detected		
TCEA1	Seq	DNA-Tumor	Mutation Not Detected		
TCF3	Seq	DNA-Tumor	Mutation Not Detected		
TCF7L2	CNA-Seq	DNA-Tumor	Amplification Not Detected		
TCF7L2	Seq	DNA-Tumor	Mutation Not Detected		
TEK	Seq	DNA-Tumor	Mutation Not Detected		
TERC	Seq	DNA-Tumor	Mutation Not Detected		
TERF2IP	Seq	DNA-Tumor	Mutation Not Detected		
TERT	Seq	DNA-Tumor	Indeterminate		
TERT	Seq	RNA-Tumor	Fusion Not Detected		
TET1	CNA-Seq	DNA-Tumor	Amplification Not Detected		
TET1	Seq	DNA-Tumor	Mutation Not Detected		
TET2	Seq	DNA-Tumor	Mutation Not Detected		
TFE3	Seq	DNA-Tumor	Mutation Not Detected		
TFE3	Seq	RNA-Tumor	Fusion Not Detected		
TFEB	Seq	DNA-Tumor	Mutation Not Detected		
TFEB	Seq	RNA-Tumor	Fusion Not Detected		
TFG	Seq	DNA-Tumor	Mutation Not Detected		
TFRC	CNA-Seq	DNA-Tumor	Amplification Not Detected		
TFRC	Seq	DNA-Tumor	Mutation Not Detected		
TGFBR1	Seq	DNA-Tumor	Indeterminate		
TGFBR2	CNA-Seq	DNA-Tumor	Amplification Not Detected		
TGFBR2	Seq	DNA-Tumor	Mutation Not Detected		
THADA	Seq	RNA-Tumor	Fusion Not Detected		
THRAP3	Seq	DNA-Tumor	Mutation Not Detected		
TIPARP	Seq	DNA-Tumor	Mutation Not Detected		
TLX3	Seq	DNA-Tumor	Mutation Not Detected		
TMB	Seq	DNA-Tumor	Low	4 Mutations/Mb	0
TMEM127	Seq	DNA-Tumor	Mutation Not Detected		
TMPRSS2	Seq	RNA-Tumor	Fusion Not Detected		
TNF	Seq	DNA-Tumor	Mutation Not Detected		
TNFAIP3	CNA-Seq	DNA-Tumor	Amplification Not Detected		
TNFAIP3	Seq	DNA-Tumor	Mutation Not Detected		
TNFRSF14	CNA-Seq	DNA-Tumor	Amplification Not Detected		
TNFRSF14	Seq	DNA-Tumor	Mutation Not Detected		
TOP1	CNA-Seq	DNA-Tumor	Amplification Not Detected		
TOP1	Seq	DNA-Tumor	Mutation Not Detected		
TOP2A	Seq	DNA-Tumor	Mutation Not Detected		
TOP3A	Seq	DNA-Tumor	Mutation Not Detected		
TOP3B	Seq	DNA-Tumor	Mutation Not Detected		
TP53	CNA-Seq	DNA-Tumor	Amplification Not Detected		
TP53	Seq	DNA-Tumor	Pathogenic Variant	Exon 7 | p.R248Q	38
TPM3	CNA-Seq	DNA-Tumor	Amplification Not Detected		
TPM3	Seq	DNA-Tumor	Mutation Not Detected		
TPM4	CNA-Seq	DNA-Tumor	Amplification Not Detected		
TPM4	Seq	DNA-Tumor	Mutation Not Detected		
TRAF3	Seq	DNA-Tumor	Mutation Not Detected		
TRAF7	Seq	DNA-Tumor	Indeterminate		
TRIM27	CNA-Seq	DNA-Tumor	Amplification Not Detected		
TRIM27	Seq	DNA-Tumor	Mutation Not Detected		
TRRAP	CNA-Seq	DNA-Tumor	Amplification Not Detected		
TRRAP	Seq	DNA-Tumor	Mutation Not Detected		
TSC1	CNA-Seq	DNA-Tumor	Amplification Not Detected		
TSC1	Seq	DNA-Tumor	Mutation Not Detected		
TSC2	CNA-Seq	DNA-Tumor	Amplification Not Detected		
TSC2	Seq	DNA-Tumor	Mutation Not Detected		
TSHR	CNA-Seq	DNA-Tumor	Amplification Not Detected		
TSHR	Seq	DNA-Tumor	Indeterminate		
TSHZ3	Seq	DNA-Tumor	Mutation Not Detected		
TYK2	Seq	DNA-Tumor	Mutation Not Detected		
TYRO3	Seq	DNA-Tumor	Indeterminate		
U2AF1	CNA-Seq	DNA-Tumor	Amplification Not Detected		
U2AF1	Seq	DNA-Tumor	Mutation Not Detected		
UBE2T	Seq	DNA-Tumor	Mutation Not Detected		
UBR5	Seq	DNA-Tumor	Mutation Not Detected		
USP6	CNA-Seq	DNA-Tumor	Amplification Not Detected		
USP6	Seq	DNA-Tumor	Mutation Not Detected		
VEGFA	Seq	DNA-Tumor	Mutation Not Detected		
VHL	Seq	DNA-Tumor	Mutation Not Detected		
VTI1A	CNA-Seq	DNA-Tumor	Amplification Not Detected		
VTI1A	Seq	DNA-Tumor	Mutation Not Detected		
WAS	Seq	DNA-Tumor	Mutation Not Detected		
WDCP	CNA-Seq	DNA-Tumor	Amplification Not Detected		
WDCP	Seq	DNA-Tumor	Mutation Not Detected		
WISP3	CNA-Seq	DNA-Tumor	Amplification Not Detected		
WISP3	Seq	DNA-Tumor	Mutation Not Detected		
WRN	CNA-Seq	DNA-Tumor	Amplification Not Detected		
WRN	Seq	DNA-Tumor	Mutation Not Detected		
WT1	CNA-Seq	DNA-Tumor	Amplification Not Detected		
WT1	Seq	DNA-Tumor	Mutation Not Detected		
WWTR1	CNA-Seq	DNA-Tumor	Amplification Not Detected		
WWTR1	Seq	DNA-Tumor	Mutation Not Detected		
XPA	Seq	DNA-Tumor	Mutation Not Detected		
XPC	CNA-Seq	DNA-Tumor	Amplification Not Detected		
XPC	Seq	DNA-Tumor	Mutation Not Detected		
XPO1	Seq	DNA-Tumor	Mutation Not Detected		
XRCC1	Seq	DNA-Tumor	Mutation Not Detected		
XRCC2	Seq	DNA-Tumor	Indeterminate		
XRCC3	Seq	DNA-Tumor	Mutation Not Detected		
YAP1	Seq	DNA-Tumor	Unclassified Variant	Exon 8 | p.G401V	8
YES1	Seq	DNA-Tumor	Mutation Not Detected		
YWHAE	CNA-Seq	DNA-Tumor	Amplification Not Detected		
YWHAE	Seq	DNA-Tumor	Mutation Not Detected		
ZBTB16	Seq	DNA-Tumor	Mutation Not Detected		
ZBTB2	Seq	DNA-Tumor	Mutation Not Detected		
ZFHX3	Seq	DNA-Tumor	Mutation Not Detected		
ZNF217	CNA-Seq	DNA-Tumor	Amplification Not Detected		
ZNF217	Seq	DNA-Tumor	Mutation Not Detected		
ZNF331	CNA-Seq	DNA-Tumor	Amplification Not Detected		
ZNF331	Seq	DNA-Tumor	Mutation Not Detected		
ZNF384	CNA-Seq	DNA-Tumor	Amplification Not Detected		
ZNF384	Seq	DNA-Tumor	Mutation Not Detected		
ZNF521	CNA-Seq	DNA-Tumor	Amplification Not Detected		
ZNF521	Seq	DNA-Tumor	Mutation Not Detected		
ZNF703	Seq	DNA-Tumor	Mutation Not Detected		
ZNRF3	Seq	DNA-Tumor	Mutation Not Detected		
ZRSR2	Seq	DNA-Tumor	Indeterminate		
